# Group A streptococcal infections in Alberta, Canada 2018–2023

**DOI:** 10.1017/S0950268824001857

**Published:** 2024-12-23

**Authors:** Gregory J. Tyrrell, Matthew Croxen, Emily McCullough, Vincent Li, Alyssa R. Golden, Irene Martin

**Affiliations:** 1 Division of Diagnostic and Applied Microbiology, Department of Laboratory Medicine and Pathology, University of Alberta, Edmonton, AB, Canada; 2 Li Ka Shing Institute of Virology, University of Alberta, Edmonton, AB, Canada; 3 Alberta Precision Laboratories – Public Health Laboratory, Edmonton, AB, Canada; 4 Women and Children’s Health Research Institute, University of Alberta, Edmonton, AB, Canada; 5 National Microbiology Laboratory, Public Health Agency of Canada, Winnipeg, MB, Canada

**Keywords:** *emm* type, genomics, invasive disease, pharyngitis, *Streptococcus pyogenes*

## Abstract

Group A streptococcal or *Streptococcus pyogenes* infections have been increasing post-COVID-19 pandemic. We describe the epidemiology of *S. pyogenes* pharyngitis and invasive disease in Alberta, Canada 2018–2023. Positive pharyngitis specimens were identified from throat swabs collected from pharyngitis patients. Invasive *S. pyogenes* was defined as the isolation of *S. pyogenes* from a normally sterile site or severe skin infection. *S. pyogenes* isolates were *emm* typed. Pharyngitis and invasive disease displayed seasonal trends preceding the COVID-19 pandemic followed by a sharp decrease during COVID-19 intervention measures. After the lifting of interventions, rates of pharyngitis and invasive disease rose. There were 182 983 positive pharyngitis specimens between 2018 and 2023 for a positivity rate of 17.6%. The highest rates occurred in the 0–9 age group in 2023 (41.5%). Invasive disease increased in 2022–2023 driven by *emm*1 and 12 types. M1_UK_ strain was the most frequent M1 type associated with invasive disease (59% of M1 isolates sequenced). Notably, out of 182 983 pharyngitis cases, there were 111 cases of invasive *S. pyogenes* detected for an invasive disease rate of 0.06%. This descriptive epidemiology of *S. pyogenes* pharyngitis and invasive *S. pyogenes* disease highlights the rapid increase in cases of *S. pyogenes* occurring in western Canada and illustrates the critical need for a vaccine.

## Introduction

Group A streptococci (*S. pyogenes*) are Gram-positive facultative anaerobic coccobacilli bacteria that grow as chains. These bacteria are responsible for a collection of different diseases in humans ranging from a mild illness such as pharyngitis (commonly referred to as strep throat) to more severe invasive diseases such as necrotizing fasciitis and toxic shock which are rare in occurrence [1, 2]. Cases of *S. pyogenes* pharyngitis tend to occur more frequently in the early stages of life (0–9 years) with some children experiencing multiple episodes of streptococcal pharyngitis [3, 4].

An important virulence factor for *S. pyogenes* is the M protein which is a long-coiled dimerized protein that projects from the Gram-positive cell wall of the bacteria [1, 5]. The M protein is encoded by the *emm* gene of which there are 261 *emm* types [6, 7]. This diversity in *emm* gene sequence results in multiple M-type proteins with some types more prevalent than others [7, 8]. Different *emm* types occur with greater frequency in low-income regions than in high-income countries where the *emm* type diversity is much less [9].

In the last 2 years (2022–2023), post-COVID-19 restrictions, the rates of *S. pyogenes* infections have significantly increased, notably in the UK and Europe as well as the USA and Australia [10-16]. Much of this increase has been driven by a small number of *S. pyogenes emm* types notably *emm*1 and *emm*12. Of the *emm*1 strains, a strain termed the M1_UK_ *S. pyogenes* strain is more prevalent in both adults and children than the previously more common M1_global_ strain [12, 17, 18]. The M1_UK_ strain is a hypervirulent *S. pyogenes* that first appeared in the UK in 2013 and subsequently spread globally [19].

The objective of this work was to describe the increase in *S. pyogenes* infections (pharyngitis and invasive disease) in Alberta, Canada from 2018 to 2023 and provide a genomic analysis of a subset of invasive *S. pyogenes* isolates identified during the increase in cases post-COVID-19 restrictions, from November 2022 to May 2023.

## Methods

### Data collection for non-invasive *S. pyogenes* pharyngitis specimens

Data on the number of pharyngitis swabs submitted to diagnostic microbiology laboratories in Alberta and the number positive for *S. pyogenes* was collated from 1 January 2018 to 31 December 2023 (72 months). This period was selected to include pre- and post-COVID-19 pandemic dates as well as overlay with the increase in invasive *S. pyogenes* infections beginning November 2022. The large data set was extracted from three different Laboratory Information Systems (LIS) used by diagnostic microbiology laboratories in Alberta during this 72-month period (LIS systems were Meditech, Cerner Millennium, and EPIC) and collated into Microsoft Excel format for analysis. Data captured included both throat cultures and molecular assays used for the detection of *S. pyogenes* from throat swabs. Alberta population estimates (used to standardize incidence calculations) were obtained from the Government of Alberta resource; http://www.ahw.gov.ab.ca/IHDA_Retrieval/ihdaData.do (accessed 1 March 2024).

### Data collection for invasive *S. pyogenes* isolates

An invasive infection caused by *S. pyogenes* is designated as a Public Health Notifiable Disease in Alberta (*https://open.alberta.ca/publications/streptococcal-disease-group-a-invasive*). Therefore, all cases are reported to public health by the laboratory identifying the case. Invasive *S. pyogenes* disease was defined as the identification of *S. pyogenes* from any sterile site including blood, brain cerebrospinal fluid, deep tissues, and joints. All invasive *S. pyogenes* isolates were identified by diagnostic microbiology laboratories in Alberta and were submitted to the APL-Public Health reference laboratory for *emm* typing and antimicrobial susceptibility assays for trending analysis.

Antibiotic susceptibility assays were performed and interpreted using reference disk diffusion methods as described by the Clinical Laboratory Standards Institute [20]. Antimicrobials assayed were penicillin, erythromycin, clindamycin, and vancomycin. All antimicrobial disks were purchased from BBL, Oxoid, England.

### Linkage of invasive *S. pyogenes* with *S. pyogenes* pharyngitis

Cases of invasive *S. pyogenes* that were identified between 1 January 2018 and 31 December 2023, were matched to *S. pyogenes* pharyngitis specimens using the personal healthcare number of each case. This was done to calculate the percentage of known *S. pyogenes* pharyngitis cases that progressed to known invasive *S. pyogenes* disease. *S. pyogenes* pharyngitis specimens were considered linked to invasive disease if the positive pharyngitis swab was collected within 7 days pre- and 7 days post-initial diagnosis of invasive *S. pyogenes* disease. This time frame was selected to capture all cases, as *S. pyogenes* pharyngitis is typically resolves within four to 5 days [21].

### *Emm* typing and whole genome sequencing

The method used to *emm* type invasive *S. pyogenes* isolates was DNA sequencing of the *emm* gene as previously described [22, 23].

For whole genome sequencing, *S. pyogenes* DNA was extracted using the MagaZorb DNA Mini-Prep Kit (Promega). Briefly, colonies grown in Todd-Hewitt Broth were centrifuged at 6000 × g for 2 minutes and the supernatant was removed. Cells were washed in 12 mM Tris and then lysed in mutanolysin/hyaluronidase lysis solution (62 ml; 10 ml 3 000 U/mL mutanolysin (Sigma), 2 mL of 30 mg/mL hyaluronidase (Sigma), and 50 mL of 10 mM Tris). Lysozyme (15 μL, 100 mg/mL; Sigma) was added and incubated for 1 h at 37 °C with shaking at 700 rpm (Eppendorf ThermoMixer F1.5). Proteinase K solution (20 μL) and RNase A (20 μL, 20 mg/mL; Qiagen or Invitrogen) were added and the tubes were incubated at room temperature for 5 minutes. ATL lysis buffer (200 μL) was added, and tubes were incubated for 2 h at 56 °C with shaking at 900 rpm (Eppendorf ThermoMixer F1.5). Extracts were centrifuged at 9000 × g for 2 minutes and wash, binding, and elution steps were completed with the KingFisher mL Purification System (Thermo Scientific) with Qiagen Buffer EB. Extracted genomic DNA was prepared using a modified Illumina DNA Prep protocol (https://www.medrxiv.org/content/10.1101/2022.02.07.22269672v1) on an Eppendorf epMotion (APL-Public Health Laboratory) or Illumina Nextera XT (National Microbiology Laboratory). Genomes were sequenced using a High Output Kit on an Illumina MiniSeq (APL-Public Health Laboratory) or an Illumina NextSeq 500/550 (National Microbiology Laboratory).

### Bioinformatic analysis

Raw sequence data quality was processed through pathogen-seq 1.0.4 (https://github.com/iaoli-dong/pathogenseq); de novo assemblies were generated with SPAdes v3.15.5 using the wrapper Shovill 1.1.0 (github.com/tseemann/shovill), with a minimum length cutoff of 300 bp [24]. In silico *emm*-typing was performed using emm-typer 0.2.0 (github.com/MDU-PHL/emmtyper), MLST performed with mlst v2.19.0 (github.com/tseemann/mlst), and virulence factor profiling using abricate 1.0.1 (github.com/tseemann/abricate) using the virulence factor database [25]. To check if any *emm*1.0 isolates were the M1_UK_ variant, assembly_snptyper v0.1.0 (github.com/boasvdp/assembly_snptyper) was used [19]. Phylogenetic trees were generated by providing the filtered core genome alignment generated by Panaroo 1.5.0 to IQ-TREE 2.2.2.7, using 1 000 ultra-fast bootstraps, 1 000 Shimodaira-Hasegawa approximate likelihood ratio tests (SH-aLRT), and ModelFinder [26, 27]. The tree was rerooted using the midroot with Gotree 0.4.3, annotated using Arcahaeopteryx 0.9930 beta (sites.google.com/view/archaeopteryx/), metadata management with csvtk 0.30.0 (github.com/shenwei356/csvtk), and ultimately visualized using GraPhlAn 1.1.3 (github.com/biobakery/graphlan) [28].

The genomic data reported in this study have been deposited in the NCBI Sequence-based Archive as part of the BioProject PRJNA1182376.

### Statistical analysis

The incidence calculation for invasive *S. pyogenes* was based on the number of isolates submitted for *emm* typing. A single isolate per case was counted unless the second isolate was collected greater than 30 days post from the first isolate. Data were graphed using OriginLab software 2023 (OriginLab Corporation, https://originlab.com).

Summary data was divided into three time periods for analysis; pre-COVID-19 years (2018–2019), years impacted by COVID-19 restrictions (2020–2022), and years’ post-COVID-19 restrictions (2023). Chi-square tests to compare study indicators between time periods were performed using R, Version 3.4.3 GUI 1.70 (2016) (The R Foundation for Statistical Computing, Vienna, Austria).

### Ethics

Ethics approval for this study was obtained from the University of Alberta Research Ethics Board (REB). Study number Pro00140378.

## Results

**Figure 1.**
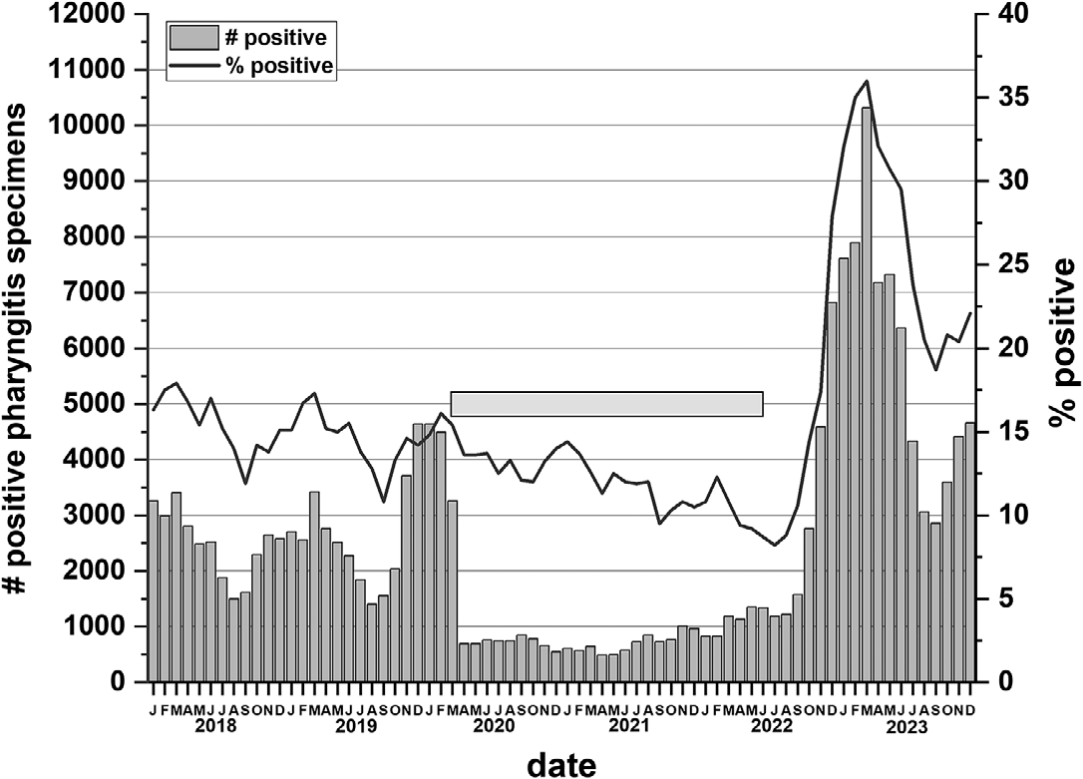
*Streptococcus pyogenes* positive specimens from cases of pharyngitis in Alberta. The columns indicate the number of positive specimens for each month over the six-year period. The line indicates the percent positivity. The horizontal gray bar indicates when Alberta imposed province wide Public Health restrictions (12 March 2020–14 June 2022). All ages are included in the data.

There were 1 041 967 pharyngitis swabs submitted for *S. pyogenes* detection over the 72-month survey period, of which 182 983 were positive for *S. pyogenes* (positivity rate of 17.6%). The number of pharyngitis swabs collected monthly varied from a low of 3 929 swabs (December 2020) to a high of 28 662 swabs (March 2023). The month with the greatest number of positive *S. pyogenes* pharyngitis swabs was March 2023 (10 321 swabs – 36.0% positivity rate) (Figure 1).

**Figure 2.**
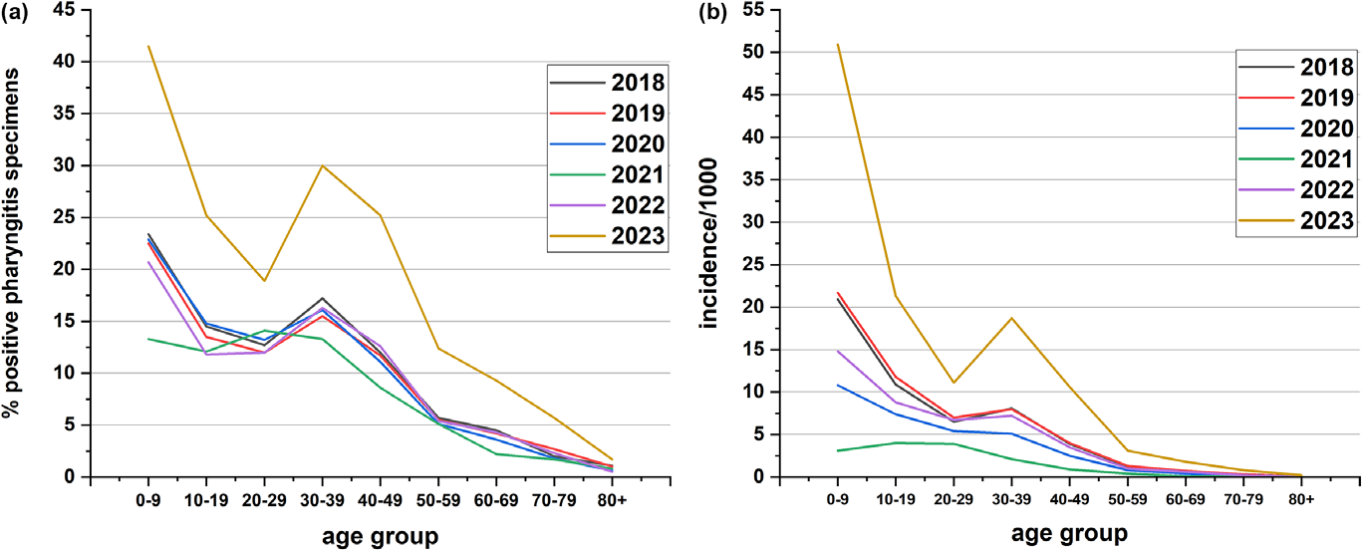
*Streptococcus pyogenes* positive pharyngitis specimens based on age and year. (a.) The percent of positive *S. pyogenes* pharyngitis from 2018 to 2023 by age group. *S. pyogenes* positivity was significantly higher in the 0–9- and 30–39-year-old age categories vs. the pooled value of all other ages (*p* < 0.0001). (b.) The incidence per 1 000 cases of *S. pyogenes* pharyngitis in Alberta from 2018 to 2023 by age group (http://www.ahw.gov.ab.ca/IHDA_Retrieval/ihdaData.do).

Overall, *S. pyogenes* positivity for pharyngitis swabs was significantly higher in 2023 vs. 2018–2019 (*p* < 0.0001). From January 2018 to March 2020 (pre-COVID-19), positive specimens for *S. pyogenes* pharyngitis for all age groups displayed seasonality peaking during winter months (December, January, and February) (Figure 1). From April 2020 to February 2022, positive *S. pyogenes* pharyngitis specimen numbers dropped to 1 000/month or fewer. The drop in submissions starting April 2020 coincided with the implementation of Public Health intervention measures mandated by the provincial government due to COVID-19 on 12 March 2020 [29]. These were lifted on 14 June 2022, approximately 2 years later (Figure 1) [30]. The age group with the greatest number of positive *S. pyogenes* pharyngitis specimens was the 0–9 years age group with 2023 having the highest annual percent positivity (41.5% (28 027/67 511)) (Figure 2a). Ages 30–39 showed the second highest positivity rate in all years except 2021, and in 2023, 30.0% of submitted specimens for this age group were positive for *S. pyogenes* (Figure 2a). For both the 0–9 and 30–39 age groups, *S. pyogenes* positivity for pharyngitis swabs was significantly higher vs. the pooled value of all other ages (*p* < 0.0001). Presenting the data as incidence per 1 000 specimens shows the 0–9-year-old age group most severely affected by *S. pyogenes* pharyngitis (Figure 2b). During COVID-19 restrictions, the years 2020 and 2021 had the lowest incidence per 1 000.

**Figure 3.**
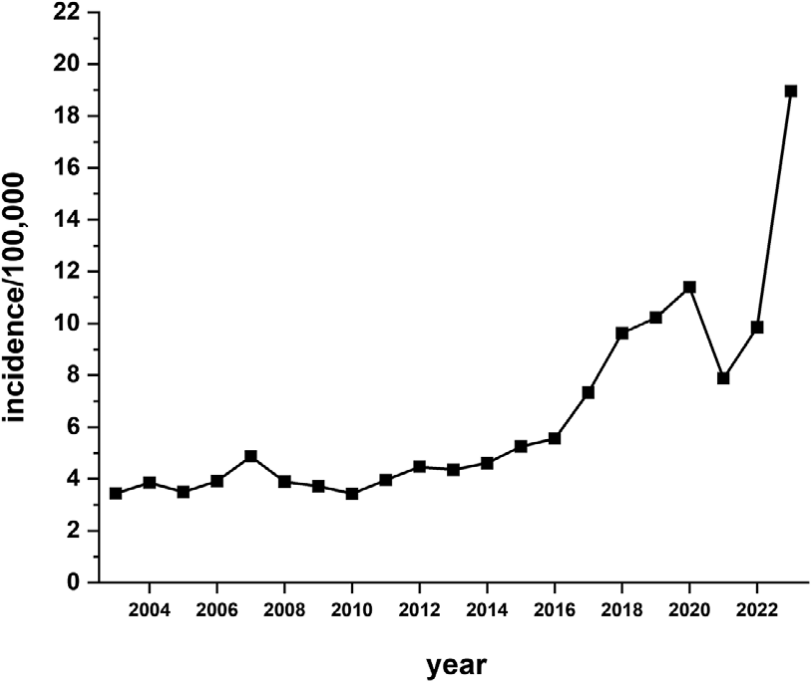
The incidence per 100 000 of invasive *Streptococcus pyogenes* disease in Alberta from 2003 to 2023 (21 years) for the general population. Incidence is based on the number of invasive *S. pyogenes* isolates submitted for *emm* typing as per notifiable disease reporting requirements. The highest incidence occurred in 2023 at 18.9/100 000.

**Figure 4.**
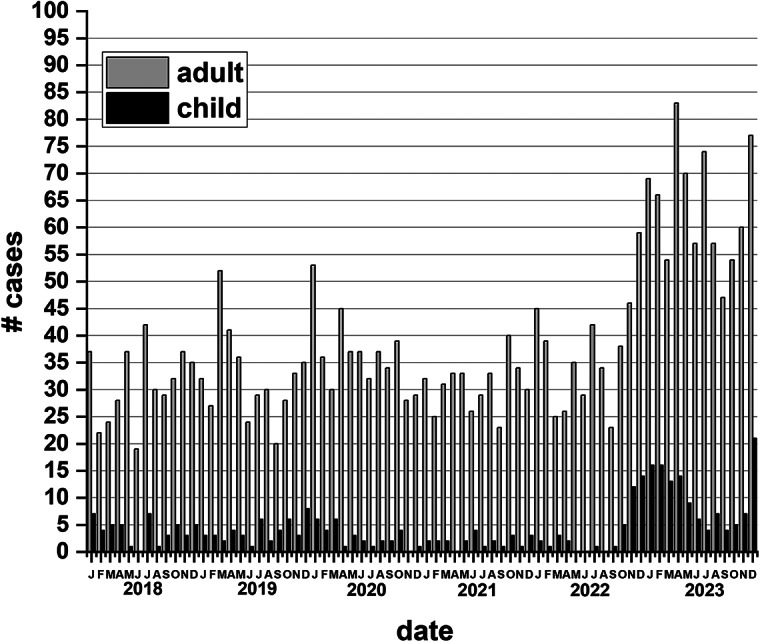
Cases of invasive *Streptococcus pyogenes* disease from 2018 to 2023 by month. Adult is defined as individuals >14 years of age. Child is defined as individuals ≤14 years of age.

The incidence of invasive *S. pyogenes* based on isolates submitted for *emm* typing showed a rise in incidence rates beginning in 2014 (4.6/100 000) with rates peaking in 2020 (11.4/100 000) and then dropping in 2021 (Figure 3). This was followed by a large increase in 2023 to 19 cases/100 000. Analysis of invasive cases from January 2018 to March 2020 (pre-pandemic) for all ages showed invasive *S. pyogenes* case numbers averaged 36.5 cases/month and from April 2020 to October 2022 (pandemic) cases averaged 34.7 cases/month. From November 2022 to December 2023 (upsurge period) this significantly increased to 72.9 cases/month (over 2-fold rise) with the greatest number of cases occurring in April 2023 (95 cases) (Figure 4.). The incidence of invasive *S. pyogenes* was significantly higher in 2023 vs. 2018–2019 for adults (>14 years of age) and children (≤14 years of age) (*p* < 0.0001). For children 14 years of age and under, the average number of cases/month was 2.5 from January 2018 to October 2022, and from November 2022 to December 2023, this increased to 12.5/month (a 5-fold increase).

**Figure 5.**
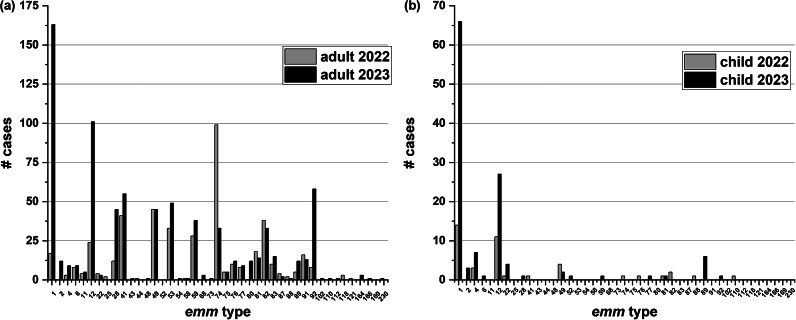
*emm* types of invasive *Streptococcus pyogenes* cases for adults and children. (a.) The number of invasive *S. pyogenes* disease by *emm* type for adults (>14 years of age) in 2022 and 2023. (b.) The *emm* types of invasive *S. pyogenes* disease for children (≤14) in 2022 and 2023. In comparison to adults, there are few cases in this age group except for *emm*1 and *emm*12.

The most frequent *emm* types in 2022 for adults were *emm*74, 49, 41, and 82. This changed in 2023 with *emm*1 and *emm*12 becoming the predominant *emm* types followed by *emm*92, *emm*41, and *emm*53 (Figure 5a). The proportion of invasive *S. pyogenes* that were *emm*1 or 12 vs. other types was significantly higher in 2023 vs. 2018–2019 (*p* < 0.0001). For children during both 2022 and 2023, most cases of invasive *S. pyogenes* were attributed to *emm*1 and *emm*12 with other *emm* types rarely seen (Figure 5b).

**Figure 6.**
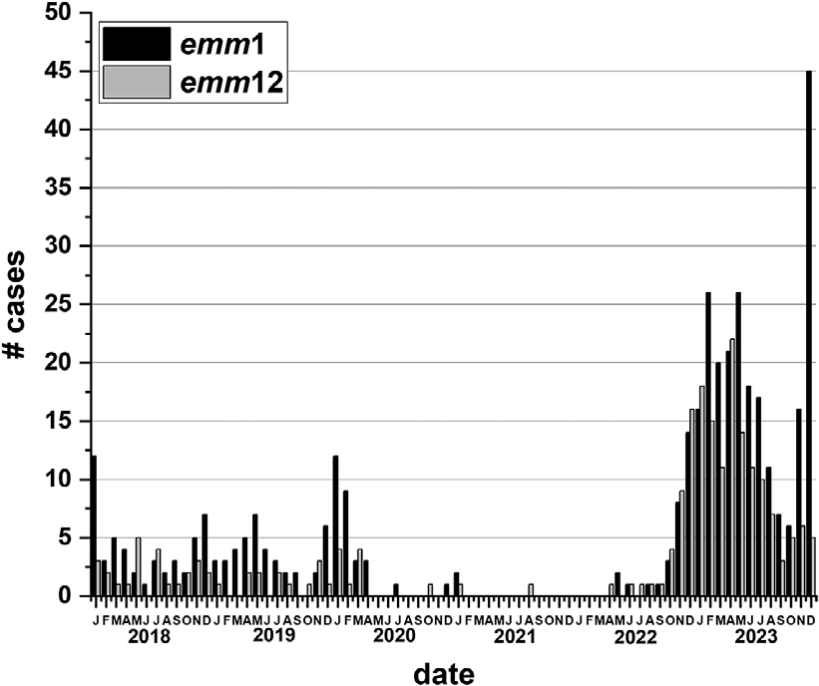
The number of cases of invasive *emm*1 and *emm*12 *Streptococcus pyogenes* from 2018 to 2023 by month. All ages are included.

During the last 2 years of the survey (2022–2023), there were 43 *emm* types identified (Figure 5a and 5b). As *emm*1 and 12 cases had increased in comparison to all other *emm* types, we took a closer look at these *emm* types over the six-year survey period. Figure 6 presents the number of cases of only *emm*1 and *emm*12. From January 2018 to April 2020 (pre-COVID-19 interventions), *emm*1 and *emm*12 averaged under five cases per month. Case numbers for *emm*1 and 12 decreased in April/May 2020 (the start of SARS-CoV-2 restriction period) and then increased sharply starting in October 2022 after the lifting of restrictions (Figure 6.).

The collection of positive *S. pyogenes* pharyngitis specimens provided us with the opportunity to identify invasive *S. pyogenes* cases that also had documented *S. pyogenes* pharyngitis. Of the 182 983 specimens of laboratory-confirmed *S. pyogenes* pharyngitis over the six-year period from 2018 to 2023, 111 cases (0.06% (60.7/100 000)) also presented with invasive *S. pyogenes* disease within 7 days of *S. pyogenes* pharyngitis diagnosis (Supplemental Table S1). Sixty-five (57.5%) of these cases were male. Thirty-three (29.5%) were associated with *emm*1 (including subtypes) and sixteen (14.3%) were associated with *emm*12 (including subtypes). Thirty-five (31.3%) of the cases with *S. pyogenes* pharyngitis and invasive *S. pyogenes* disease were 14 years of age and under. Of these 35 cases of *S. pyogenes* pharyngitis linked to invasive disease in children, 45.7% (16/35) were invasive *emm*1 (including subtypes) and 20% (7/35) were invasive *emm*12 (including subtypes) (Supplemental Table S1).

From 1 January 2018 to 31 December 2023, antimicrobial susceptibility assays were performed on 3 179 invasive *S. pyogenes* isolates (2018–442 isolates, 2019–433, 2020–469, 2021–398, 2022–519, and 2023–918). All isolates were fully susceptible to penicillin and vancomycin. Overall, erythromycin and clindamycin resistance in invasive *S. pyogenes* cases were significantly higher in 2023 vs. 2018–2019 (*p* < 0.0001). Antimicrobial resistance to erythromycin ranged from a low of 5.3% in 2021 to a high of 15.1% in 2023 (Table 1). This is similar to clindamycin with a low of 4.0% in 2021 and a high of 13.3% in 2023 (Table 1). The most common *emm* types associated with clindamycin and erythromycin resistance were *emm*92, 77, 83, and 53 (Supplemental Table S2). Together, these four *emm* types accounted for 71.4% of erythromycin-resistant isolates and 69.3% of clindamycin-resistant isolates. Of the 591 isolates of *emm*1 and 12 over the 6 years surveyed, only five were erythromycin resistant and three clindamycin resistant.

**Table 1.** Erythromycin and clindamycin resistance (%) 2018–2023

Year	Erythromycin	Clindamycin	Total # isolates tested
2018	8.1%	7.9%	442
2019	6.0%	6.7%	433
2020	8.5%	6.8%	469
2021	5.3%	4.0%	398
2022	8.7%	7.7%	519
2023	15.1%	14.6%	878

Erythromycin and clindamycin resistance was significantly higher in 2023 vs. 2018–2019 (*p* < 0.0001).

**Figure 7.**
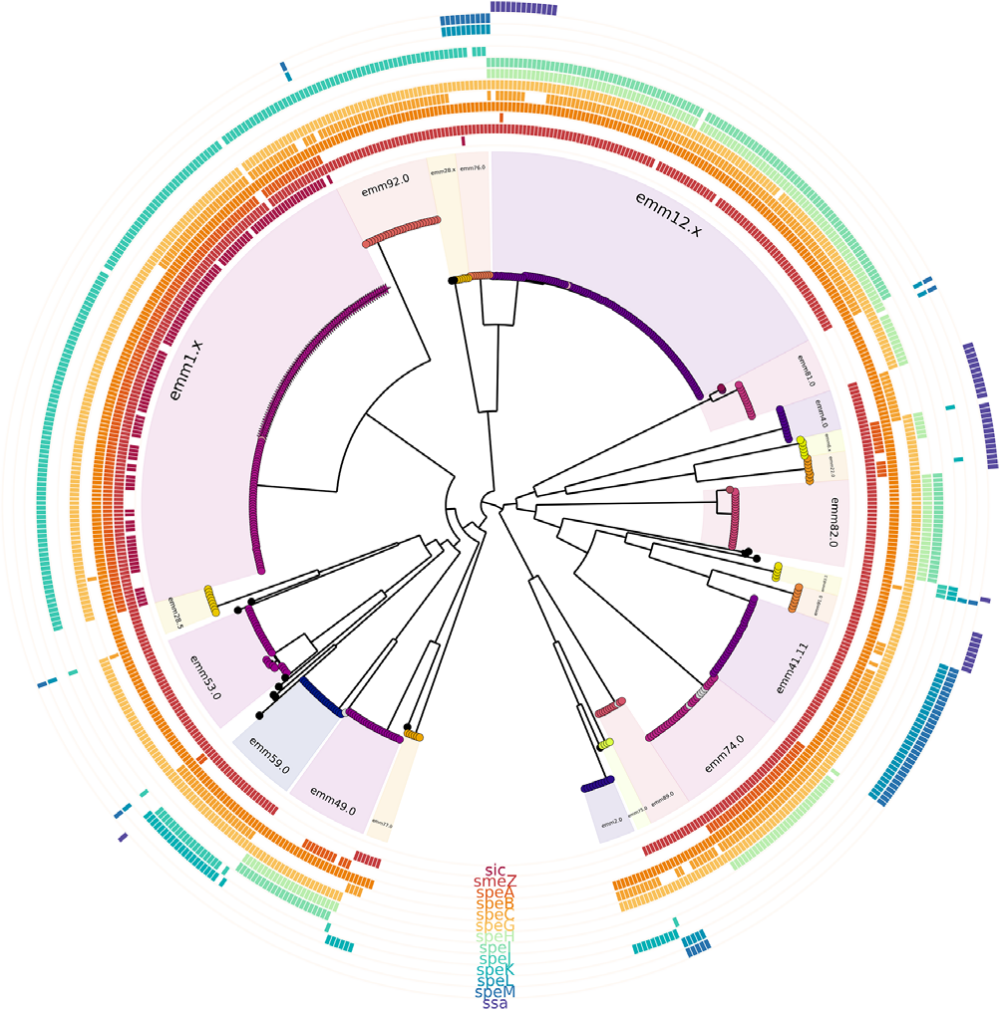
Phylogenetic tree analysis of 549 invasive *Streptococcus pyogenes* isolates from November 2022 to May 2023. A maximum likelihood phylogenetic tree was constructed from the core genomes using a GTR + F + I + G4 model. *Emm* types are indicated, sequence types are coloured by nodes, and the M1_UK_ variant is indicated by a star-shaped node. Bars in concentric circles represent the presence of the sic gene followed by 12 different exotoxin genes found in *S. pyogenes.*

**Figure 8.**
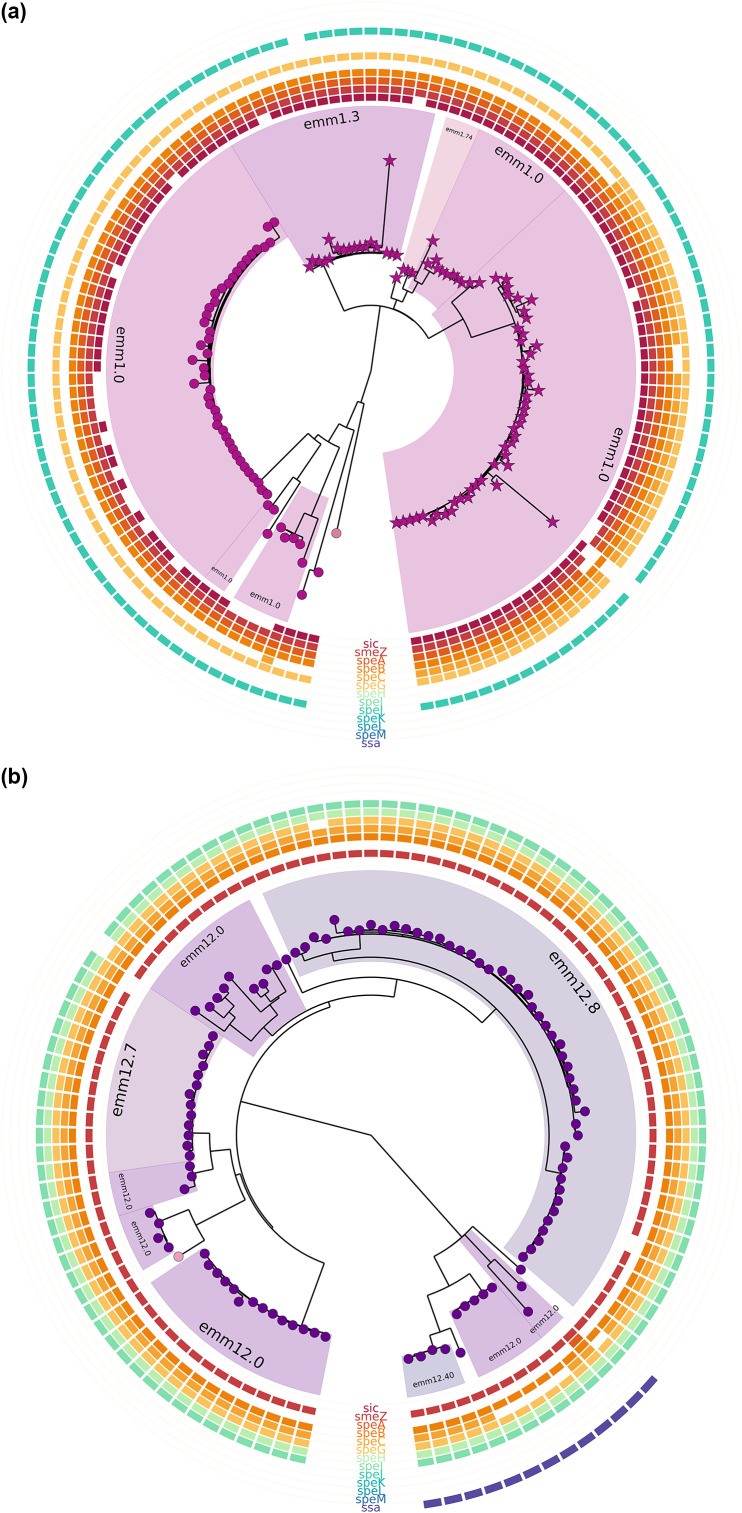
Phylogenetic trees of (a) 134 *emm*1 invasive *Streptococcus pyogenes* isolates and (b) 106 invasive *emm*12 isolates all collected from November 2022 to May 2023. Maximum likelihood phylogenetic trees constructed with core gene alignment using a HYK + F + I model for *emm*1 and K3Pu + F + I model for *emm*12. (a). The phylogenetic tree shows 54 M1_global_ and 80 M1_UK_ *S. pyogenes* isolates. Sequence types are coloured by nodes, and the M1_UK_ variant is indicated by a star-shaped node. Bars in concentric circles represent the presence of *sic* gene followed by 12 different exotoxin genes found in *S. pyogenes.* (b). Phylogenetic tree of *emm*12 isolates in Alberta.

Genome sequencing was completed for 549 invasive *S. pyogenes* isolates collected from 1 November 2022 to 31 May 2023 (all *emm* types) (Figure 7 and Supplemental Table S3). There were 192 *speA* isolates (35.0%), 301 *speC* isolates (54∙8%), 80 isolates with both *speA* and *speC* (14.6%), and 302 with *spd1* (55.0%). Of the 134 *emm*1 isolates sequenced, the genomic analysis showed 79 isolates belonged to the M1_UK_ lineage based on 27 single nucleotide variants in the core genome (59.0% of *emm*1 cases) and 54 isolates belonged to the M1_global_ strain (40.3%) with one case as M1_intermediate_ (Figure 7 and Supplemental Table S3). Ninety-eight percent of M1 isolates possessed the *speA* gene whereas two isolates, (an M1_UK_ and an M1_intermediate_) did not (Figure 8a and Supplemental Table S3). The *speC* gene was present in 33.6% of M1 isolates. Interestingly, the *speC* gene was present in a distinct branch of the M1_UK_ group and absent in all M1_global_ isolates except for one isolate (Figure 8a and Supplemental Table S3). The *emm*12 *S. pyogenes* isolates exhibited higher diversity in comparison to the *emm*1 stains (Figure 8b). Only 19 isolates of the 549 sequenced had all three *ssa*, *speC*, and *spd1* genes, (three *emm*12.0, four *emm*12.4, 11 *emm*4.0, and one *emm*58.0) (Supplemental Table S3).

## Discussion

The data presented for pharyngitis specimens over the 6 years surveyed showed yearly fluctuating changes in positivity rates for *S. pyogenes.* Prior to the implementation of Public Health interventions for COVID-19, pharyngeal *S. pyogenes* positivity rates showed a predictable seasonality trend as previously reported by others with the highest rates occurring in winter months and lower in summer [4, 31, 32]. The implementation of Public Health interventions for COVID-19 reduced these rates significantly starting in 2020 [29]. It was after these restrictions were lifted that pharyngitis *S. pyogenes* positivity rates sharply increased peaking in March 2023 [30]. This should not be surprising as these interventions were targeted towards the respiratory transmission of SARS-CoV-2, which is also a similar transmission route for *S. pyogenes.* It is unusual though to have such a large increase in cases as it could have been predicted that case numbers would have returned to pre-COVID-19 levels once restrictions were lifted.

Breaking down *S. pyogenes* pharyngitis by age group showed specimens were the most prevalent in the 0–9-year-old age group. A study by Mponponsuo et al., analyzed cases of *S. pyogenes* pharyngitis in Calgary, Alberta from 2010 to 2018 [3]. This study included 1 074 154 tests, of which 16.6% were positive for *S. pyogenes*, similar to our study. These investigators found the 5–14 age group had the highest positivity rate (42.2%) [3]. In a recent study by Kline et al., this group found that the 0–4 and 5–9 age groups also had the most frequent *S. pyogenes* pharyngitis visits to healthcare providers of all ages [4]. It is concerning that for the 0–9-year-old age group in our study, rates approached nearly 50% positivity, a very high rate of *S. pyogenes* pharyngitis. A possible reason for this sharp rise in *S. pyogenes* pharyngitis may include more mixing of this age group as COVID-19 restrictions were lifted and children returned to daycares and schools.

The increase in *S. pyogenes* pharyngitis specimens was mirrored by increases in *S. pyogenes* invasive disease. The incidence of invasive disease rose sharply from 9.8/100 000 in 2022 to 19/100 000 in 2023, a significant increase. It should be noted that 2023 was not the first year that the incidence of invasive *S. pyogenes* disease began to increase. The incidence of invasive *S. pyogenes* started to rise in 2014, peaking in 2020. However, the magnitude of the increase over this six-year period was not at the same level as the increase in 2023. The increase in 2023 reflects increases in invasive *S. pyogenes* disease seen elsewhere such as the UK, other countries in Europe, the USA, and Australia [10, 14, 33, 34]. The reasons for this steady climb in invasive disease since 2014 are not completely clear and potentially involve several factors. These may include the introduction of new strains, greater vulnerability in the population, and lifting of COVID-19 restrictions leading to increases in population density. Examples include educational institutions resuming in-class instruction and removal of the requirement for masking in areas such as shopping locations.

Prior to the SARS-CoV-2 pandemic, *emm*1 and 12 were the most frequent *emm* types associated with invasive disease in Alberta, along with other *emm* types [35]. During the COVID-19 restriction period, these two *emm* types almost disappeared as *S. pyogenes* bacteria responsible for invasive disease. Once restrictions were lifted, both *emm* types returned in increased prevalence as the predominant *emm* types and at higher rates compared to rates pre-COVID-19. Reasons for the *emm*1 and *emm*12 resurgence are likely multifactorial including lifting of Public Health interventions thereby allowing the potential for increased respiratory transmission of these *emm* types. It is interesting that both *emm*1 and 12 are part of the A-C cluster (*emm*1:A-C3 and *emm*12:A-C4), which is considered a cluster associated with the throat as opposed to *emm* type clusters associated more with cutaneous disease [36]. The only other A-C *emm* cluster type found in Alberta from cases of invasive *S. pyogenes* was *emm*3, however, invasive disease caused by this *emm* type has not occurred to the same extent as *emm*1 and *emm*12 for reasons which are not well understood.

The collection of *S. pyogenes* pharyngitis data provided an opportunity to determine the rate of *S. pyogenes* pharyngitis progressing to invasive disease for the Alberta population. For every 100 000 positive *S. pyogenes* pharyngitis specimens, approximately 61 cases progressed into invasive disease over the six-year period surveyed. This is a crude estimate of the risk of developing invasive disease in patients with pharyngitis as several events occurring during the survey period could have affected the results. A major event was the SARS-CoV-2 pandemic as it is likely individuals may have not sought *S. pyogenes* pharyngitis testing due to adherence to isolation requirements. This coupled with an apparent decrease in *S. pyogenes* transmission during pandemic years may have skewed the estimate during the pandemic. Also, physicians may not have collected a specimen for a laboratory diagnosis and alternatively prescribed based on symptoms. It should also be noted that pharmacies in Alberta can perform point-of-care tests for *S. pyogenes* pharyngitis. Those cases of *S. pyogenes* pharyngitis diagnosed in pharmacies are not captured in the patient’s provincial health record. While these and other variables may affect our estimate of invasive disease occurring during an episode of pharyngitis, an average of 61 cases of invasive disease per every 100 000 cases of pharyngitis over a six-year period we believe is plausible.

Both erythromycin and clindamycin resistance in Alberta for invasive *S. pyogenes* ranged from 4–9% from 2018 to 2022 however, in 2023, rates significantly increased to between 14–16%. These rates are higher than what we have previously reported for Canada (2018–2022) and likely reflect regional differences in circulating strains in Alberta [37]. Similar to our Alberta study, predominant resistant *emm* types, *emm*11, 53, 77, 83, and 92, also have been reported as significant erythromycin-resistant *emm* types in Canada and the USA [37, 38]. It is interesting that *emm*92 is the most frequently encountered erythromycin/clindamycin resistant *emm* type as this *emm* type has been shown to be associated with erythromycin/clindamycin resistant *emm*92 strains in adult IV drug users in the USA [39]. Efforts are now being made to determine the demographics of the *emm*92 iStrep A isolates in our survey study.

During the global increase in iStrep A disease, much interest has focused on the M1_UK_ *S. pyogenes* bacteria as it rapidly expands throughout the world replacing M1_global_ as the predominant M1 type in just a few short years [11, 40-42]. M1_UK_ was first detected in Alberta in 2016 and is now broadly distributed across Canada [37, 43]. For the *S. pyogenes* isolates for which we sequenced (November 2022 to May 2023), M1_UK_ accounted for close to 60% of the invasive M1 isolates making it the predominant *emm* type strain over this period. Past reports have shown that M1_UK_ produces high levels of the SpeA exotoxin in comparison to the M1_global_ strain [19]. Both SpeA and SpeC exotoxin have been shown to be associated with increased fitness and virulence of *S. pyogenes* strains causing disease [19, 44, 45]. All M1 isolates for which genomic sequencing was completed possessed the *speA* toxin gene except for two M1_UK_ isolates. The *speC* gene was less frequent in the M1 isolates.

In summary, rates of both *S. pyogenes* pharyngitis and invasive *S. pyogenes* disease have substantially increased in Alberta, Canada post-COVID-19. This increase has persisted for over a year since SARS-CoV-2 restrictions were lifted. Invasive disease rates have been driven by predominately two *emm* types, *emm*1 and *emm*12 with M1_UK_ more frequent than M1_global_.

## Supporting information

Tyrrell et al. supplementary material Tyrrell et al. supplementary material

## Data Availability

The data that support the findings of this study are available on request from the corresponding author. Restrictions may apply to the availability of personal data linked to patient information.

## Supplementary material

For supplementary material accompanying this paper visit http://doi.org/10.1017/S0950268824001857. click here to view supplementary material
