# Supplementary material for: Group A streptococcal infections in Alberta, Canada 2018–2023
Source: Epidemiol Infect. 2024 Dec 23;153:e35. doi: 10.1017/S0950268824001857 (PMC11869071; doi:10.1017/S0950268824001857)
Supplement: Tyrrell et al. supplementary material [file S0950268824001857sup001.docx]

**Epidemiology and Infection**

**Group A streptococcal infections in Alberta, Canada 2018-2023.**

Gregory J. Tyrrell, Matthew Croxen. Emily McCullough, Vincent Li, Alyssa R. Golden, Irene Martin

Supplementary Material

Supplementary Table S1: Cases of invasive *S. pyogenes* with laboratory confirmed *S. pyogenes* pharyngitis 7 days pre or post

invasive disease diagnosis.

Supplementary Table S2: The *emm* types of erythromycin and clindamycin resistant invasive *S. pyogenes* isolates; 2018-2023.

Supplementary Table S3: Table of the 549 invasive *S. pyogenes* isolates for genomic sequencing was completed.

**Supplementary Table S1: Cases of invasive *S. pyogenes* with laboratory confirmed *S. pyogenes* pharyngitis 7 days pre or post**

**invasive disease diagnosis.** Data was collected for 2018-2023.

| **case #** | **year** | **age** | **gender** | **invasive source** | **#days between + throat and invasive disease** | ***emm* type** |
| --- | --- | --- | --- | --- | --- | --- |
| 1 | 2018 | 1 | M | Pleural Fluid | 0 | 3.1 |
| 2 | 2018 | 2 | F | Blood | -1 | 1 |
| 3 | 2018 | 2 | F | Neck | 4 | 28 |
| 4 | 2018 | 3 | F | Blood | -1 | 28 |
| 5 | 2018 | 15 | F | Blood | 3 | 1 |
| 6 | 2018 | 28 | M | Blood | 0 | 1 |
| 7 | 2018 | 29 | F | Blood | 0 | 74 |
| 8 | 2018 | 29 | M | Throat | 0 | 89 |
| 9 | 2018 | 30 | M | Blood | 0 | 4 |
| 10 | 2018 | 31 | F | Blood | 0 | 41 |
| 11 | 2018 | 31 | M | Blood | 4 | 53 |
| 12 | 2018 | 44 | M | Blood | 0 | 22 |
| 13 | 2018 | 49 | M | Neck | 1 | 81 |
| 14 | 2018 | 51 | M | Blood | 0 | 1 |
| 15 | 2018 | 55 | M | Blood | 0 | 9 |
| 16 | 2018 | 64 | M | Blood | 0 | 2 |
| 17 | 2018 | 67 | M | Blood | 0 | 93 |
| 18 | 2018 | 76 | M | Blood | 0 | 81 |
| 19 | 2019 | 4 | M | Blood | 1 | 1 |
| 20 | 2019 | 4 | M | Blood | 1 | 49 |
| 21 | 2019 | 8 | M | Blood | -1 | 12.8 |
| 22 | 2019 | 14 | F | Leg | 5 | 1 |
| 23 | 2019 | 49 | M | Peritonsillar | 1 | 1 |
| 24 | 2019 | 62 | M | Blood | 0 | 76 |
| 25 | 2019 | 81 | F | Blood | 0 | 28 |
| 26 | 2020 | 6 | F | Blood | 0 | 1 |
| 27 | 2020 | 7 | M | Blood | 0 | 1 |
| 28 | 2020 | 21 | M | Blood | -1 | 11 |
| 29 | 2020 | 34 | F | Blood | 0 | 3.1 |
| 30 | 2020 | 36 | F | Blood | 0 | 1 |
| 31 | 2020 | 38 | M | Blood | 0 | 22 |
| 32 | 2020 | 39 | F | Blood | 2 | 12.8 |
| 33 | 2020 | 46 | M | Blood | 5 | 49 |
| 34 | 2020 | 49 | F | Throat | 0 | 1 |
| 35 | 2020 | 64 | F | Knee | 3 | 2 |
| 36 | 2021 | 11 | F | Tissue Other | 6 | 76 |
| 37 | 2021 | 12 | F | Throat | 0 | 49 |
| 38 | 2021 | 13 | F | Throat | 0 | 49 |
| 39 | 2021 | 16 | M | Leg tissue | 6 | 77 |
| 40 | 2021 | 30 | F | Blood | 0 | 89 |
| 41 | 2021 | 37 | M | Blood | 0 | 49 |
| 42 | 2021 | 41 | M | Other | 2 | 49 |
| 43 | 2021 | 48 | F | Throat | 0 | 49 |
| 44 | 2021 | 52 | F | Throat | 0 | 49 |
| 45 | 2021 | 57 | M | Blood | 0 | 49 |
| 46 | 2021 | 67 | F | Neck | 1 | 89 |
| 47 | 2022 | 5 | M | Blood | 0 | 1 |
| 48 | 2022 | 5 | M | Palm | 6 | 1.3 |
| 49 | 2022 | 6 | M | Blood | 1 | 12.8 |
| 50 | 2022 | 7 | M | Blood | 0 | 1 |
| 51 | 2022 | 8 | F | Blood | 0 | 12.7 |
| 52 | 2022 | 29 | M | Tissue Other | 0 | 1 |
| 53 | 2022 | 30 | F | Throat | 0 | 91 |
| 54 | 2022 | 32 | M | Blood | 1 | 49 |
| 55 | 2022 | 33 | F | Blood | -1 | 87 |
| 56 | 2022 | 34 | F | Throat | 0 | 12.7 |
| 57 | 2022 | 36 | M | Neck | 2 | 82 |
| 58 | 2022 | 38 | M | Blood | 1 | 1 |
| 59 | 2022 | 40 | M | Blood | 0 | 53 |
| 60 | 2022 | 40 | F | Blood | 0 | 1.3 |
| 61 | 2022 | 40 | F | Blood | 0 | 76 |
| 62 | 2022 | 43 | M | Blood | 0 | 12.8 |
| 63 | 2022 | 46 | F | Blood | 0 | 74 |
| 64 | 2022 | 56 | F | Blood | 1 | 76 |
| 65 | 2022 | 59 | M | Blood | 0 | 74 |
| 66 | 2023 | 1 | M | Swab | 1 | 1 |
| 67 | 2023 | 1 | F | Blood | 0 | 1 |
| 68 | 2023 | 1 | M | Blood | 3 | 12.8 |
| 69 | 2023 | 1 | M | Shoulder | 3 | 12.8 |
| 70 | 2023 | 2 | M | Blood | 0 | 4 |
| 71 | 2023 | 4 | M | Neck | 0 | 12 |
| 72 | 2023 | 4 | M | Blood | 0 | 1 |
| 73 | 2023 | 5 | F | Blood | 0 | 2 |
| 74 | 2023 | 5 | F | Throat | 0 | 4 |
| 75 | 2023 | 6 | F | Blood | 0 | 1 |
| 76 | 2023 | 6 | F | Blood | 0 | 1 |
| 77 | 2023 | 6 | M | Blood | 0 | 89 |
| 78 | 2023 | 6 | F | Blood | 0 | 1 |
| 79 | 2023 | 7 | M | Blood | -1 | 12.4 |
| 80 | 2023 | 7 | F | Blood | 0 | 1 |
| 81 | 2023 | 9 | M | Tonsil, Left | 1 | 4 |
| 82 | 2023 | 10 | M | Pharynx | 0 | 1 |
| 83 | 2023 | 17 | M | Pleural fluid | 4 | 1 |
| 84 | 2023 | 23 | M | Throat | -1 | 87 |
| 85 | 2023 | 28 | M | Neck aspirate | 5 | 73 |
| 86 | 2023 | 29 | M | Blood | 0 | 1 |
| 87 | 2023 | 31 | F | Blood | 0 | 82 |
| 88 | 2023 | 32 | F | Blood | 1 | 1.29 |
| 89 | 2023 | 32 | F | Blood | 0 | 4 |
| 90 | 2023 | 35 | F | Blood | 0 | 49 |
| 91 | 2023 | 35 | F | Blood | 0 | 12.8 |
| 92 | 2023 | 36 | F | Blood | -1 | 12.8 |
| 93 | 2023 | 37 | M | Ear | 2 | 12.8 |
| 94 | 2023 | 38 | F | Neck | 2 | 2 |
| 95 | 2023 | 39 | M | Neck | 2 | 1 |
| 96 | 2023 | 42 | M | Blood | 0 | 41.11 |
| 97 | 2023 | 44 | M | Blood | 0 | 1 |
| 98 | 2023 | 47 | M | Blood | 0 | 49 |
| 99 | 2023 | 50 | M | Foot, Left | 0 | 1 |
| 100 | 2023 | 52 | F | Blood | 6 | 11 |
| 101 | 2023 | 53 | M | Blood | 0 | 1 |
| 102 | 2023 | 53 | M | Blood | 5 | 41.11 |
| 103 | 2023 | 56 | M | Wound arm | 0 | 1 |
| 104 | 2023 | 58 | M | Blood | 0 | 49 |
| 105 | 2023 | 59 | F | Blood | 0 | 59 |
| 106 | 2023 | 60 | F | Blood | 0 | 4 |
| 107 | 2023 | 62 | M | Blood | -2 | 49 |
| 108 | 2023 | 62 | M | Blood | 0 | 12 |
| 119 | 2023 | 65 | M | Neck aspirate | 0 | 92 |
| 110 | 2023 | 66 | M | Blood | 0 | 59 |
| 111 | 2023 | 71 | M | Blood | -1 | 12.7 |

**Supplementary Table S2: The *emm* types of erythromycin and clindamycin resistant invasive *S. pyogenes* isolates; 2018-2023**

| ***emm* type** | **erythromycin R** | **%** | **clindamycin R** | **%** |
| --- | --- | --- | --- | --- |
| 1 | 2 | 0.6% | 1 | 0.4% |
| 11 | 29 | 9.3% | 29 | 10.6% |
| 12 | 3 | 1.0% | 2 | 0.7% |
| 28 | 1 | 0.3% | 0 | 0.0% |
| 4 | 7 | 2.3% | 8 | 2.9% |
| 44 | 1 | 0.3% | 0 | 0.0% |
| 48 | 2 | 0.6% | 2 | 0.7% |
| 49 | 2 | 0.6% | 2 | 0.7% |
| **53** | **34** | **10.9%** | **34** | **12.4%** |
| 58 | 2 | 0.6% | 1 | 0.4% |
| 59 | 2 | 0.6% | 2 | 0.7% |
| 68 | 5 | 1.6% | 2 | 0.7% |
| 69 | 0 | 0.0% | 1 | 0.4% |
| 73 | 1 | 0.3% | 1 | 0.4% |
| 74 | 2 | 0.6% | 2 | 0.7% |
| 76 | 6 | 1.9% | 7 | 2.6% |
| **77** | **57** | **18.7%** | **48** | **17.5%** |
| 78 | 1 | 0.3% | 1 | 0.4% |
| 81 | 5 | 1.6% | 5 | 1.8% |
| **83** | **41** | **13.2%** | **35** | **12.8%** |
| 87 | 2 | 0.6% | 3 | 1.1% |
| 88 | 1 | 0.3% | 1 | 0.4% |
| 89 | 4 | 1.3% | 4 | 1.5% |
| 9 | 3 | 1.0% | 3 | 1.1% |
| **92** | **89** | **28.6%** | **73** | **26.6%** |
| 94 | 2 | 0.6% | 1 | 0.4% |
| 102 | 3 | 1.0% | 3 | 1.1% |
| 110 | 1 | 0.3% | 1 | 0.4% |
| 112 | 1 | 0.3% | 0 | 0.0% |
| 169 | 2 | 0.6% | 2 | 0.7% |
| **Total # isolates** | **311** |  | **274** |  |

**Supplementary Table S3:** Table of the 549 invasive *S. pyogenes* isolates for genomic sequencing was completed.

| **num-id** | **month-year collected** | **Provlab-number** | **mlst** | **emm-type** | **m1uk-status** | **sic** | **smeZ** | **speB** | **speA** | **speC** | **speG** | **speH** | **speI** | **speJ** | **speK** | **speL** | **speM** | **ssa** | **spd1** |
| --- | --- | --- | --- | --- | --- | --- | --- | --- | --- | --- | --- | --- | --- | --- | --- | --- | --- | --- | --- |
| 1 | 11/22 | SS-22-0001248 | 28 | EMM1.0 | M1global | 93.2 | 100 | 100 | 100 | . | 100 | . | . | 100 | . | . | . | . | . |
| 2 | 11/22 | SS-22-0001251 | 334 | EMM82.0 | N/A | . | 100 | 100 | . | . | 100 | 100 | 100 | . | . | . | . | . | . |
| 3 | 11/22 | SS-22-0001258 | 39 | EMM4.0 | N/A | . | 100 | 100 | . | 100 | . | . | . | . | . | . | . | 100 | 100 |
| 4 | 11/22 | SS-22-0001259 | 39 | EMM4.0 | N/A | . | 100 | 100 | . | 100 | . | . | . | . | . | . | . | 100 | 100 |
| 5 | 11/22 | SS-22-0001262 | 101 | EMM89.0 | N/A | . | 100 | 100 | . | . | 100 | . | . | . | . | . | . | . | . |
| 6 | 11/22 | SS-22-0001263 | 347 | EMM53.0 | N/A | . | 100 | 100 | . | . | 100 | . | . | . | . | . | . | . | . |
| 7 | 11/22 | SS-22-0001266 | 150 | EMM75.0 | N/A | . | 99.57 | 100 | . | . | 100 | . | . | . | . | 100 | 100 | . | . |
| 8 | 11/22 | SS-22-0001268 | 28 | EMM1.74 | M1UK | 90.61 | 100 | 100 | 100 | . | 100 | . | . | 100 | . | . | . | . | . |
| 9 | 11/22 | SS-22-0001270 | 36 | EMM12.8 | N/A | . | 100 | 100 | . | 100 | 100 | 100 | 100 | . | . | . | . | . | 100 |
| 10 | 11/22 | SS-22-0001273 | 28 | EMM1.0 | M1UK | 90.61 | 100 | 100 | 100 | 100 | 100 | . | . | 100 | . | . | . | . | 100 |
| 11 | 11/22 | SS-22-0001278 | 12 | EMM91.0 | N/A | . | 100 | 100 | . | . | 100 | . | . | . | . | . | . | 100 | . |
| 12 | 11/22 | SS-22-0001279 | 853 | EMM83.1 | N/A | . | 100 | 100 | . | . | 100 | . | . | . | . | . | . | . | . |
| 13 | 11/22 | SS-22-0001281 | 50 | EMM76.0 | N/A | . | 100 | 100 | . | . | 100 | . | . | 100 | . | 100 | 100 | . | . |
| 14 | 11/22 | SS-22-0001286 | 63 | EMM77.0 | N/A | . | 99.86 | 100 | . | 100 | . | . | . | . | 97.56 | . | . | . | 100 |
| 15 | 11/22 | SS-22-0001295 | 579 | EMM41.11 | N/A | . | 100 | 100 | . | 100 | 100 | . | . | . | . | 100 | 100 | . | 100 |
| 16 | 11/22 | SS-22-0001297 | 1176 | EMM28.5 | N/A | . | 100 | 100 | . | . | . | . | . | . | . | . | . | . | . |
| 17 | 11/22 | SS-22-0001303 | 909 | EMM81.0 | N/A | . | . | 100 | . | . | 100 | 100 | . | . | . | . | . | . | . |
| 18 | 11/22 | SS-22-0001305 | 50 | EMM76.0 | N/A | . | 100 | 100 | . | . | 100 | . | . | 100 | . | 100 | 100 | . | . |
| 19 | 11/22 | SS-22-0001312 | - | EMM74.0 | N/A | . | 100 | 100 | 100 | 100 | 100 | 98.73 | . | . | . | . | . | . | 100 |
| 20 | 11/22 | SS-22-0001317 | 28 | EMM1.0 | M1global | . | 100 | 100 | 100 | . | 100 | . | . | 100 | . | . | . | . | . |
| 21 | 11/22 | SS-22-0001321 | 63 | EMM77.0 | N/A | . | 99.86 | 100 | . | 100 | . | . | . | . | 97.56 | . | . | . | 100 |
| 22 | 11/22 | SS-22-0001332 | 120 | EMM74.0 | N/A | . | 100 | 100 | 100 | 100 | 100 | 98.73 | . | . | . | . | . | . | 100 |
| 23 | 11/22 | SS-22-0001337 | 36 | EMM12.8 | N/A | . | 100 | 100 | . | 100 | 100 | 100 | 100 | . | . | . | . | . | 100 |
| 24 | 11/22 | SS-22-0001343 | 101 | EMM89.0 | N/A | . | 100 | 100 | . | 100 | 100 | . | . | . | . | . | . | . | 100 |
| 25 | 11/22 | SS-22-0001346 | 172 | EMM59.0 | N/A | . | 100 | 100 | . | 100 | 100 | . | . | 100 | . | . | . | . | 100 |
| 26 | 11/22 | SS-22-0001349 | 36 | EMM12.0 | N/A | . | 100 | 100 | . | 100 | 100 | 100 | 100 | . | . | . | . | . | 100 |
| 27 | 11/22 | SS-22-0001350 | 624 | EMM81.0 | N/A | . | . | 100 | . | . | 100 | 100 | . | . | . | . | . | . | . |
| 28 | 11/22 | SS-22-0001352 | 579 | EMM41.11 | N/A | . | 100 | 100 | . | 100 | 100 | . | . | . | . | 100 | 100 | . | 100 |
| 29 | 11/22 | SS-22-0001353 | 36 | EMM12.7 | N/A | . | 100 | 100 | . | 100 | 100 | 100 | 100 | . | . | . | . | . | 100 |
| 30 | 11/22 | SS-22-0001355 | 28 | EMM1.0 | M1global | . | 100 | 100 | 100 | . | 100 | . | . | 100 | . | . | . | . | . |
| 31 | 11/22 | SS-22-0001363 | 36 | EMM12.0 | N/A | . | 100 | 100 | . | 100 | 100 | 100 | 100 | . | . | . | . | . | 100 |
| 32 | 11/22 | SS-22-0001365 | 28 | EMM1.0 | M1UK | 90.61 | 100 | 100 | 100 | 100 | 100 | . | . | 100 | . | . | . | . | 100 |
| 33 | 11/22 | SS-22-0001367 | 1040 | EMM121.2 | N/A | . | 99.43 | 100 | . | 100 | 100 | . | . | 100 | . | 100 | 100 | . | 100 |
| 34 | 11/22 | SS-22-0001375 | 50 | EMM76.0 | N/A | . | 100 | 100 | . | . | 100 | . | . | 100 | . | 100 | 100 | . | . |
| 35 | 11/22 | SS-22-0001378 | 82 | EMM92.0 | N/A | . | 100 | 100 | . | 100 | 100 | . | . | 100 | . | . | . | . | 100 |
| 36 | 11/22 | SS-22-0001382 | 433 | EMM49.0 | N/A | . | . | 100 | . | . | 100 | 100 | 100 | . | . | . | . | . | . |
| 37 | 11/22 | SS-22-0001383 | 909 | EMM81.0 | N/A | . | . | 100 | . | . | 100 | 100 | . | . | . | . | . | . | . |
| 38 | 11/22 | SS-22-0001395 | 579 | EMM41.11 | N/A | . | 100 | 100 | . | 100 | 100 | . | . | . | . | 100 | 100 | . | 100 |
| 39 | 11/22 | SS-22-0001401 | 36 | EMM12.8 | N/A | . | 100 | 100 | . | 100 | 100 | 100 | 100 | . | . | . | . | . | 100 |
| 40 | 11/22 | SS-22-0001408 | 28 | EMM1.3 | M1UK | 90.61 | 100 | 100 | 100 | . | 100 | . | . | 100 | . | . | . | . | . |
| 41 | 11/22 | SS-22-0001417 | 433 | EMM49.0 | N/A | . | . | 100 | . | . | 100 | 100 | 100 | . | . | . | . | . | . |
| 42 | 11/22 | SS-22-0001420 | 63 | EMM77.0 | N/A | . | 99.86 | 100 | . | 100 | . | . | . | . | 97.56 | . | . | . | 100 |
| 43 | 11/22 | SS-22-0001426 | 82 | EMM92.0 | N/A | . | 100 | 100 | . | 100 | 100 | . | . | 100 | . | . | . | . | 100 |
| 44 | 11/22 | SS-22-0001431 | 36 | EMM12.8 | N/A | . | 100 | 100 | . | 100 | 100 | 100 | 100 | . | . | . | . | . | 100 |
| 45 | 11/22 | SS-22-0001432 | 12 | EMM91.0 | N/A | . | 100 | 100 | . | . | 100 | . | . | . | . | . | . | 100 | . |
| 46 | 11/22 | SS-22-0001433 | 82 | EMM92.0 | N/A | . | 100 | 100 | . | 100 | 100 | . | . | 100 | . | . | . | . | 100 |
| 47 | 11/22 | SS-22-0001435 | 192 | EMM25.1 | N/A | . | 100 | 100 | . | . | 100 | . | . | . | . | . | . | . | . |
| 48 | 11/22 | SS-22-0001436 | 46 | EMM22.0 | N/A | . | 100 | 100 | . | . | 100 | . | . | . | . | . | . | 100 | . |
| 49 | 11/22 | SS-22-0001454 | 46 | EMM22.0 | N/A | . | 100 | 100 | 100 | . | 100 | . | . | . | . | . | . | 100 | . |
| 50 | 11/22 | SS-22-0001458 | 39 | EMM4.0 | N/A | . | 100 | 100 | . | 100 | . | . | . | . | . | . | . | 100 | 100 |
| 51 | 11/22 | SS-22-0001463 | 36 | EMM12.8 | N/A | . | 100 | 100 | . | 100 | 100 | 100 | 100 | . | . | . | . | . | 100 |
| 52 | 11/22 | SS-22-0001468 | 36 | EMM12.7 | N/A | . | 100 | 100 | . | 100 | 100 | 100 | 100 | . | . | . | . | . | 100 |
| 53 | 11/22 | SS-22-0001472 | 28 | EMM1.0 | M1UK | 90.61 | 100 | 100 | 100 | 100 | 100 | . | . | 100 | . | . | . | . | 100 |
| 54 | 11/22 | SS-22-0001482 | 12 | EMM91.0 | N/A | . | 100 | 100 | . | . | 100 | . | . | . | . | . | . | 100 | . |
| 55 | 11/22 | SS-22-0001484 | 120 | EMM74.0 | N/A | . | 100 | 100 | 100 | 100 | 100 | 98.73 | . | . | . | . | . | . | 100 |
| 56 | 11/22 | SS-22-0001485 | 172 | EMM59.0 | N/A | . | 100 | 100 | . | 100 | 100 | . | . | 100 | 100 | . | . | . | 100 |
| 57 | 11/22 | SS-22-0001489 | 120 | EMM74.0 | N/A | . | 100 | 100 | 100 | 100 | 100 | 98.73 | . | . | . | . | . | . | 100 |
| 58 | 11/22 | SS-22-0001494 | 579 | EMM41.11 | N/A | . | 100 | 100 | . | 100 | 100 | . | . | . | . | 100 | 100 | . | 100 |
| 59 | 12/22 | SS-22-0001477 | 909 | EMM81.0 | N/A | . | . | 100 | . | . | 100 | 100 | . | . | . | . | . | . | . |
| 60 | 12/22 | SS-22-0001483 | 579 | EMM41.11~ | N/A | . | 100 | 100 | . | . | 100 | . | . | . | . | 100 | 100 | . | . |
| 61 | 12/22 | SS-22-0001495 | 36 | EMM12.0 | N/A | . | 100 | 100 | . | . | 100 | 100 | 100 | . | . | . | . | 100 | . |
| 62 | 12/22 | SS-22-0001504 | 579 | EMM41.11 | N/A | . | 100 | 100 | . | 100 | 100 | . | . | . | . | 100 | 100 | . | 100 |
| 63 | 12/22 | SS-22-0001507 | 205 | EMM230.0 | N/A | . | 100 | 100 | . | 100 | 100 | . | . | 100 | 100 | 100 | 100 | . | 100 |
| 64 | 12/22 | SS-22-0001511 | 28 | EMM1.0 | M1global | . | 100 | 100 | 100 | . | 100 | . | . | 100 | . | . | . | . | . |
| 65 | 12/22 | SS-22-0001513 | 347 | EMM53.0 | N/A | . | 100 | 100 | . | . | 100 | . | . | . | . | . | . | . | . |
| 66 | 12/22 | SS-22-0001517 | 36 | EMM12.8 | N/A | . | 100 | 100 | . | 100 | 100 | 100 | 100 | . | . | . | . | . | 100 |
| 67 | 12/22 | SS-22-0001519 | 62 | EMM87.0 | N/A | . | 100 | 100 | . | . | 100 | . | . | 100 | 97.56 | . | . | 100 | . |
| 68 | 12/22 | SS-22-0001525 | 36 | EMM12.0 | N/A | . | 100 | 100 | . | . | 100 | 100 | 100 | . | . | . | . | 100 | . |
| 69 | 11/22 | SS-22-0001527 | 347 | EMM53.0 | N/A | . | 100 | 100 | . | . | 100 | . | . | . | . | . | . | . | . |
| 70 | 12/22 | SS-22-0001528 | 28 | EMM1.0 | M1global | 92.23 | 100 | 100 | 100 | . | 100 | . | . | 100 | . | . | . | . | . |
| 71 | 12/22 | SS-22-0001530 | 36 | EMM12.0 | N/A | . | 100 | 100 | . | 100 | 100 | 100 | 100 | . | . | . | . | . | 100 |
| 72 | 12/22 | SS-22-0001534 | 28 | EMM1.3 | M1UK | 90.61 | 100 | 100 | 100 | . | 100 | . | . | 100 | . | . | . | . | . |
| 73 | 12/22 | SS-22-0001537 | 433 | EMM49.0 | N/A | . | . | 100 | . | . | 100 | 100 | 100 | . | . | . | . | . | . |
| 74 | 12/22 | SS-22-0001538 | 579 | EMM41.11 | N/A | . | 100 | 100 | . | 100 | 100 | . | . | . | . | 100 | 100 | . | 100 |
| 75 | 12/22 | SS-22-0001539 | 28 | EMM1.0 | M1global | . | 100 | 100 | 100 | . | 100 | . | . | 100 | . | . | . | . | . |
| 76 | 12/22 | SS-22-0001545 | 36 | EMM12.0 | N/A | . | 100 | 100 | . | 100 | 100 | 100 | 100 | . | . | . | . | . | 100 |
| 77 | 12/22 | SS-22-0001546 | 579 | EMM41.11 | N/A | . | 100 | 100 | . | 100 | 100 | . | . | . | . | 100 | 100 | . | 100 |
| 78 | 12/22 | SS-22-0001548 | 458 | EMM28.0 | N/A | . | 100 | 100 | . | 100 | 100 | . | . | 100 | . | . | . | . | 100 |
| 79 | 12/22 | SS-22-0001549 | 334 | EMM82.0 | N/A | . | 100 | 100 | . | . | 100 | 100 | 100 | . | . | . | . | . | . |
| 80 | 12/22 | SS-22-0001552 | 63 | EMM77.0 | N/A | . | 99.86 | 100 | . | 100 | . | . | . | . | 97.56 | . | . | . | 100 |
| 81 | 12/22 | SS-22-0001553 | 28 | EMM1.0 | M1UK | 90.61 | 100 | 100 | 100 | 100 | 100 | . | . | 100 | . | . | . | . | 100 |
| 82 | 12/22 | SS-22-0001554 | 458 | EMM28.0 | N/A | . | 100 | 100 | . | 100 | 100 | . | . | 100 | . | . | . | . | 100 |
| 83 | 12/22 | SS-22-0001564 | 120 | EMM74.0 | N/A | . | 100 | 100 | 100 | 100 | 100 | 98.73 | . | . | . | . | . | . | 100 |
| 84 | 12/22 | SS-22-0001565 | 347 | EMM53.0 | N/A | . | 100 | 100 | . | . | 100 | . | . | . | . | . | . | . | . |
| 85 | 12/22 | SS-22-0001566 | 28 | EMM1.0 | M1global | . | 100 | 100 | 100 | . | 100 | . | . | 100 | . | . | . | . | . |
| 86 | 12/22 | SS-22-0001568 | 39 | EMM4.0 | N/A | . | 100 | 100 | . | 100 | . | . | . | . | . | . | . | 100 | 100 |
| 87 | 12/22 | SS-22-0001569 | 579 | EMM41.11 | N/A | . | 100 | 100 | . | 100 | 100 | . | . | . | . | 100 | 100 | . | 100 |
| 88 | 12/22 | SS-22-0001570 | 36 | EMM12.8 | N/A | . | 100 | 100 | . | 100 | 100 | 100 | 100 | . | . | . | . | . | 100 |
| 89 | 12/22 | SS-22-0001571 | 36 | EMM12.0 | N/A | . | 100 | 100 | . | 100 | 100 | 100 | 100 | . | . | . | . | . | 100 |
| 90 | 12/22 | SS220001575 | 36 | EMM12.8 | N/A | . | 100 | 100 | . | 100 | 100 | 100 | 100 | . | . | . | . | . | 100 |
| 91 | 12/22 | SS220001585 | 28 | EMM1.0 | M1UK | 90.61 | 100 | 100 | 100 | . | 100 | . | . | 100 | . | . | . | . | . |
| 92 | 12/22 | SS220001586 | 167 | EMM118.0~ | N/A | . | 100 | 100 | . | 100 | 100 | . | . | . | . | . | . | . | 100 |
| 93 | 12/22 | SS220001689 | 36 | EMM12.0 | N/A | . | 100 | 100 | . | 100 | 100 | 100 | 100 | . | . | . | . | . | 100 |
| 94 | 12/22 | SS220001592 | 579 | EMM41.11 | N/A | . | 100 | 100 | . | 100 | 100 | . | . | . | . | 100 | 100 | . | 100 |
| 95 | 12/22 | SS220001595 | 334 | EMM82.0 | N/A | . | 100 | 100 | . | . | 100 | 100 | 100 | . | . | . | . | . | . |
| 96 | 12/22 | SS220001601 | 28 | EMM1.3 | M1UK | 90.61 | 100 | 100 | 100 | . | 100 | . | . | 100 | . | . | . | . | . |
| 97 | 12/22 | SS220001602 | 28 | EMM1.0 | M1global | . | 100 | 100 | 100 | . | 100 | . | . | 100 | . | . | . | . | . |
| 98 | 12/22 | SS220001604 | 579 | EMM41.11 | N/A | . | 100 | 100 | . | 100 | 100 | . | . | . | . | 100 | 100 | . | 100 |
| 99 | 12/22 | SS220001605 | 334 | EMM82.0 | N/A | . | 100 | 100 | . | . | 100 | 100 | 100 | . | . | . | . | . | . |
| 100 | 12/22 | SS220001606 | 36 | EMM12.0 | N/A | . | 100 | 100 | . | 100 | 100 | 100 | 100 | . | . | . | . | . | 100 |
| 101 | 12/22 | SS220001610 | 347 | EMM53.0 | N/A | . | 100 | 100 | . | . | 100 | . | . | . | . | . | . | . | . |
| 102 | 12/22 | SS220001613 | 334 | EMM82.0 | N/A | . | 100 | 100 | . | . | 100 | 100 | 100 | . | . | . | . | . | . |
| 103 | 12/22 | SS220001616 | 28 | EMM1.0 | M1UK | 90.61 | 100 | 100 | 100 | . | 100 | . | . | 100 | . | . | . | . | . |
| 104 | 12/22 | SS220001617 | 909 | EMM81.0 | N/A | . | . | 100 | . | . | 100 | 100 | . | . | . | . | . | . | . |
| 105 | 12/22 | SS220001618 | 579 | EMM41.11~ | N/A | . | 100 | 100 | . | 100 | 100 | . | . | . | . | 100 | 100 | . | 100 |
| 106 | 12/22 | SS220001619 | 28 | EMM1.0 | M1global | . | 100 | 100 | 100 | . | 100 | . | . | 100 | . | . | . | . | . |
| 107 | 12/22 | SS220001621 | 176 | EMM58.0 | N/A | . | 98.72 | 100 | . | 100 | 100 | . | . | . | 97.56 | . | . | 100 | 100 |
| 108 | 12/22 | SS220001622 | 853 | EMM83.1 | N/A | . | 100 | 100 | . | . | 100 | . | . | . | . | . | . | . | . |
| 109 | 12/22 | SS220001625 | 28 | EMM1.74 | M1UK | 90.61 | 100 | 100 | 100 | . | 100 | . | . | 100 | . | . | . | . | . |
| 110 | 12/22 | SS220001628 | 36 | EMM12.8 | N/A | . | 100 | 100 | . | 100 | 100 | 100 | 100 | . | . | . | . | . | 100 |
| 111 | 12/22 | SS220001632 | 36 | EMM12.0 | N/A | . | 100 | 100 | . | 100 | 100 | 100 | 100 | . | . | . | . | . | 100 |
| 112 | 12/22 | SS220001636 | 909 | EMM81.0 | N/A | . | . | 100 | . | . | 100 | 100 | . | . | . | . | . | . | . |
| 113 | 12/22 | SS220001637 | 12 | EMM91.0 | N/A | . | 100 | 100 | . | . | 100 | . | . | . | . | . | . | 100 | . |
| 114 | 12/22 | SS220001642 | 120 | EMM74.0~ | N/A | . | 100 | 100 | 100 | 100 | 100 | 98.73 | . | . | . | . | . | . | 100 |
| 115 | 12/22 | SS220001643 | 82 | EMM92.0 | N/A | . | 100 | 100 | . | 100 | 100 | . | . | 100 | . | . | . | . | 100 |
| 116 | 12/22 | SS220001646 | 36 | EMM12.0 | N/A | . | 100 | 100 | . | 100 | 100 | 100 | 100 | . | . | . | . | . | 100 |
| 117 | 12/22 | SS220001650 | 172 | EMM59.0 | N/A | . | 100 | 100 | . | 100 | 100 | . | . | 100 | 100 | . | . | . | 100 |
| 118 | 12/22 | SS220001651 | 433 | EMM49.0 | N/A | . | . | 100 | 100 | . | 100 | 100 | 100 | . | . | . | . | . | . |
| 119 | 12/22 | SS220001652 | 28 | EMM1.3 | M1UK | 90.61 | 100 | 100 | 100 | . | 100 | . | . | 100 | . | . | . | . | . |
| 120 | 12/22 | SS220001653 | 120 | EMM74.0 | N/A | . | 100 | 100 | 100 | 100 | 100 | 98.73 | . | . | . | . | . | . | 100 |
| 121 | 12/22 | SS220001657 | 36 | EMM12.7 | N/A | . | 100 | 100 | . | 100 | 100 | 100 | 100 | . | . | . | . | . | 100 |
| 122 | 12/22 | SS220001658 | - | EMM89.0 | N/A | . | 100 | 100 | . | 100 | 100 | . | . | . | . | . | . | . | 100 |
| 123 | 12/22 | SS220001666 | 36 | EMM12.0 | N/A | . | 100 | 100 | . | 100 | 100 | 100 | 100 | . | . | . | . | . | 100 |
| 124 | 12/22 | SS220001667 | 36 | EMM12.0 | N/A | . | 100 | 100 | 100 | 100 | 100 | 100 | 100 | . | . | . | . | 100 | 100 |
| 125 | 12/22 | SS220001668 | 52 | EMM28.0 | N/A | . | 100 | 100 | . | 100 | 100 | . | . | 100 | . | . | . | . | 100 |
| 126 | 12/22 | SS220001669 | 347 | EMM53.0 | N/A | . | 100 | 100 | . | . | 100 | . | . | . | . | . | . | . | . |
| 127 | 12/22 | SS220001671 | 82 | EMM92.0~ | N/A | . | 100 | 100 | . | 100 | 100 | . | . | 100 | . | . | . | . | 100 |
| 128 | 12/22 | SS220001672 | 28 | EMM1.0 | M1UK | 90.61 | 100 | 100 | 100 | 100 | 100 | . | . | 100 | . | . | . | . | 100 |
| 129 | 12/22 | SS220001678 | 579 | EMM41.11~ | N/A | . | 100 | 100 | . | 100 | 100 | . | . | . | . | 100 | 100 | . | 100 |
| 130 | 12/22 | SS220001679 | 28 | EMM1.0 | M1UK | 90.61 | 100 | 100 | 100 | 100 | 100 | . | . | 100 | . | . | . | . | 100 |
| 131 | 12/22 | SS220001683 | 36 | EMM12.0 | N/A | . | 100 | 100 | . | 100 | 100 | 100 | 100 | . | . | . | . | . | 100 |
| 132 | 12/22 | SS220001685 | 347 | EMM53.0 | N/A | . | 100 | 100 | . | . | 100 | . | . | . | . | . | . | . | . |
| 133 | 12/22 | SS220001688 | 82 | EMM92.0 | N/A | . | 100 | 100 | . | 100 | 100 | . | . | 100 | . | . | . | . | 100 |
| 134 | 12/22 | SS220001690 | 172 | EMM59.0 | N/A | . | 100 | 100 | . | 100 | 100 | . | . | 100 | 100 | . | . | . | 100 |
| 135 | 01/23 | SS230000008 | 382 | EMM6.4 | N/A | . | 100 | 100 | 100 | . | 100 | 98.73 | . | . | . | . | . | 100 | . |
| 136 | 01/23 | SS230000010 | 50 | EMM76.0 | N/A | . | 100 | 100 | . | . | 100 | . | . | 100 | . | 100 | 100 | . | . |
| 137 | 01/23 | SS230000012 | 101 | EMM89.0 | N/A | . | 100 | 100 | . | 100 | 100 | . | . | . | . | . | . | . | 100 |
| 138 | 01/23 | SS230000014 | 50 | EMM76.0 | N/A | . | 100 | 100 | . | . | 100 | . | . | 100 | . | 100 | 100 | . | . |
| 139 | 01/23 | SS230000016 | 579 | EMM41.11 | N/A | . | 100 | 100 | . | 100 | 100 | . | . | . | . | 100 | 100 | . | 100 |
| 140 | 01/23 | SS230000020 | 55 | EMM2.0 | N/A | . | . | 100 | . | 100 | 100 | . | . | . | 100 | . | . | . | 100 |
| 141 | 01/23 | SS230000022 | 347 | EMM53.0 | N/A | . | 100 | 100 | . | . | 100 | . | . | . | . | . | . | . | . |
| 142 | 01/23 | SS230000023 | 347 | EMM53.0 | N/A | . | 100 | 100 | . | . | 100 | . | . | . | . | . | . | . | . |
| 143 | 01/23 | SS230000029 | 39 | EMM4.0 | N/A | . | 98.43 | 100 | . | 100 | . | . | . | . | . | . | . | 100 | 99.34 |
| 144 | 01/23 | SS230000031 | 28 | EMM1.0 | M1UK | 90.61 | 100 | 100 | 100 | 100 | 100 | . | . | 100 | . | . | . | . | 100 |
| 145 | 01/23 | SS230000032 | 36 | EMM12.0 | N/A | . | 100 | 100 | . | 100 | 100 | 100 | 100 | . | . | . | . | . | 100 |
| 146 | 01/23 | SS230000034 | 36 | EMM12.8 | N/A | . | 100 | 100 | . | 100 | 100 | 100 | 100 | . | . | . | . | . | 100 |
| 147 | 01/23 | SS230000040 | 28 | EMM1.0 | M1global | . | 100 | 100 | 100 | . | 100 | . | . | 100 | . | . | . | . | . |
| 148 | 01/23 | SS230000041 | 120 | EMM74.0 | N/A | . | 100 | 100 | 100 | 100 | 100 | 98.73 | . | . | . | . | . | . | 100 |
| 149 | 01/23 | SS230000050 | 28 | EMM1.0 | M1global | . | 100 | 100 | 100 | . | 100 | . | . | 100 | . | . | . | . | . |
| 150 | 01/23 | SS230000058 | 36 | EMM12.7 | N/A | . | 100 | 100 | . | 100 | 90.21 | 100 | 100 | . | . | . | . | . | 100 |
| 151 | 01/23 | SS230000061 | 909 | EMM81.0 | N/A | . | . | 100 | . | . | 100 | 100 | . | . | . | . | . | . | . |
| 152 | 01/23 | SS230000067 | 120 | EMM74.0 | N/A | . | 100 | 100 | 100 | 100 | 100 | 98.73 | . | . | . | . | . | . | 100 |
| 153 | 01/23 | SS230000076 | 50 | EMM76.0 | N/A | . | 100 | 100 | . | . | 100 | . | . | 100 | . | 100 | 100 | . | . |
| 154 | 01/23 | SS230000078 | 28 | EMM1.0 | M1global | . | 100 | 100 | 99.73 | . | 100 | . | . | 100 | . | . | . | . |  |
| 155 | 01/23 | SS230000080 | 36 | EMM12.7 | N/A | . | 100 | 100 | . | 100 | 100 | 100 | 100 | . | . | . | . | . | 100 |
| 156 | 01/23 | SS230000082 | 28 | EMM1.0 | M1global | . | 100 | 100 | 100 | . | 100 | . | . | 100 | . | . | . | . | . |
| 157 | 01/23 | SS230000084 | 120 | EMM74.0~ | N/A | . | 100 | 100 | 100 | 100 | 100 | 98.73 | . | . | . | . | . | . | 100 |
| 158 | 01/23 | SS230000086 | 46 | EMM22.0 | N/A | . | 100 | 100 | 100 | . | 100 | . | . | . | . | . | . | 100 | . |
| 159 | 01/23 | SS230000087 | 28 | EMM1.0 | M1global | . | 100 | 100 | 100 | . | 100 | . | . | 100 | . | . | . | . | . |
| 160 | 01/23 | SS230000090 | 36 | EMM12.8 | N/A | . | 100 | 100 | . | 100 | 100 | 100 | 100 | . | . | . | . | . | 100 |
| 161 | 01/23 | SS230000096 | 36 | EMM12.0 | N/A | . | 100 | 100 | . | 100 | 100 | 100 | 100 | . | . | . | . | 100 | 100 |
| 162 | 01/23 | SS230000097 | 433 | EMM49.0 | N/A | . | . | 100 | 100 | . | 100 | 100 | 100 | . | . | . | . | . | . |
| 163 | 01/23 | SS230000107 | 853 | EMM83.1 | N/A | . | 87.46 | 100 | . | . | 100 | . | . | . | . | . | . | . | . |
| 164 | 01/23 | SS230000108 | 36 | EMM12.8 | N/A | . | 100 | 100 | . | 100 | 100 | 100 | 100 | . | . | . | . | . | 100 |
| 165 | 01/23 | SS230000109 | - | EMM74.0~ | N/A | . | 100 | 100 | 100 | 100 | 100 | 98.73 | . | . | . | . | . | . | 100 |
| 166 | 01/23 | SS230000112 | 382 | EMM6.4 | N/A | . | 100 | 100 | 100 | . | 100 | 98.73 | . | . | . | . | . | 100 | . |
| 167 | 01/23 | SS230000114 | 579 | EMM41.11~ | N/A | . | 100 | 100 | . | . | 100 | . | . | . | . | 100 | 100 | . | . |
| 168 | 01/23 | SS230000115 | 28 | EMM1.0 | M1UK | 100 | 100 | 100 | 100 | 100 | 100 | . | . | 100 | . | . | . | . | 100 |
| 169 | 01/23 | SS230000116 | 347 | EMM53.0 | N/A | . | 100 | 100 | . | . | 100 | . | . | . | . | . | . | . | . |
| 170 | 01/23 | SS230000118 | 28 | EMM1.3 | M1UK | 90.61 | 100 | 100 | 100 | . | 100 | . | . | 100 | . | . | . | . | . |
| 171 | 01/23 | SS230000120 | 172 | EMM59.0 | N/A | . | 100 | 100 | . | 100 | 100 | . | . | 100 | 100 | . | . | . | 100 |
| 172 | 01/23 | SS230000121 | 28 | EMM1.0 | M1global | 93.2 | 100 | 100 | 100 | . | 100 | . | . | 100 | . | . | . | . | . |
| 173 | 01/23 | SS230000122 | 909 | EMM81.0 | N/A | . | . | 100 | . | . | 100 | . | . | . | . | 100 | 100 | . | . |
| 174 | 01/23 | SS230000124 | 433 | EMM49.0 | N/A | . | . | 100 | 100 | . | 100 | 100 | 100 | . | . | . | . | . | . |
| 175 | 01/23 | SS230000128 | 101 | EMM89.0 | N/A | . | 94.3 | 100 | . | 100 | 100 | . | . | . | . | . | . | . | 100 |
| 176 | 01/23 | SS230000129 | 36 | EMM12.7 | N/A | . | 93.59 | 100 | . | 100 | 100 | 100 | 100 | . | . | . | . | . | 100 |
| 177 | 01/23 | SS230000130 | 120 | EMM74.0 | N/A | . | 100 | 100 | 100 | 100 | 100 | 98.73 | . | . | . | . | . | . | 100 |
| 178 | 01/23 | SS230000132 | 347 | EMM53.0 | N/A | . | 100 | 100 | . | . | 100 | . | . | . | . | . | . | . | . |
| 179 | 01/23 | SS230000136 | 36 | EMM12.40 | N/A | . | 100 | 100 | . | 100 | 100 | 100 | 100 | . | . | . | . | 100 | 100 |
| 180 | 01/23 | SS230000137 | 36 | EMM12.7 | N/A | . | 100 | 100 | . | 100 | 100 | 100 | 100 | . | . | . | . | . | 100 |
| 181 | 01/23 | SS230000143 | 55 | EMM2.0 | N/A | . | . | 100 | . | 100 | 100 | . | . | . | 100 | . | . | . | 100 |
| 182 | 01/23 | SS230000145 | 36 | EMM12.0 | N/A | . | 100 | 100 | . | 100 | 100 | 100 | 100 | . | . | . | . | . | 100 |
| 183 | 01/23 | SS230000149 | 172 | EMM59.0 | N/A | . | 100 | 100 | . | 100 | 100 | . | . | 100 | 100 | . | . | . | 100 |
| 184 | 01/23 | SS230000151 | 433 | EMM49.0 | N/A | . | . | 100 | . | . | 100 | 100 | 100 | . | . | . | . | . | . |
| 185 | 01/23 | SS230000152 | 82 | EMM92.0 | N/A | . | 90.31 | 100 | . | 100 | 91.21 | . | . | 100 | . | . | . | . | 100 |
| 186 | 01/23 | SS230000154 | 28 | EMM1.3 | M1UK | 100 | 100 | 100 | 100 | . | 100 | . | . | 100 | . | . | . | . | . |
| 187 | 01/23 | SS230000157 | 36 | EMM12.0 | N/A | . | 100 | 100 | . | 100 | 100 | 100 | 100 | . | . | . | . | . | 100 |
| 188 | 01/23 | SS230000158 | 55 | EMM2.0 | N/A | . | . | 100 | . | 100 | 100 | . | . | . | 100 | . | . | . | 100 |
| 189 | 01/23 | SS230000160 | 82 | EMM92.0 | N/A | . | 94.3 | 100 | . | 100 | 100 | . | . | 100 | . | . | . | . | 100 |
| 190 | 01/23 | SS-23-0000162 | 28 | EMM1.0 | M1UK | 100 | 100 | 100 | 100 | 100 | 100 | . | . | 100 | . | . | . | . | 100 |
| 191 | 01/23 | SS-23-0000163 | 39 | EMM4.0 | N/A | . | 100 | 100 | . | 100 | . | . | . | . | . | . | . | 100 | 100 |
| 192 | 01/23 | SS-23-0000164 | 433 | EMM49.0 | N/A | . | . | 100 | . | . | 100 | 100 | 100 | . | . | . | . | . | . |
| 193 | 01/23 | SS-23-0000165 | 36 | EMM12.8~ | N/A | . | 100 | 100 | . | 100 | 100 | 100 | 100 | . | . | . | . | . | 100 |
| 194 | 01/23 | SS-23-0000166 | 172 | EMM59.0 | N/A | . | 100 | 100 | . | 100 | 100 | . | . | 100 | 100 | . | . | . | 100 |
| 195 | 01/23 | SS-23-0000174 | 46 | EMM22.0 | N/A | . | 100 | 100 | 100 | . | 100 | . | . | . | . | . | . | 100 | . |
| 196 | 01/23 | SS-23-0000175 | 28 | EMM1.3 | M1UK | 100 | 100 | 100 | 100 | . | 100 | . | . | 100 | . | . | . | . | . |
| 197 | 01/23 | SS-23-0000176 | 28 | EMM1.0 | M1global | 93.2 | 100 | 100 | 100 | . | 100 | . | . | 100 | . | . | . | . | . |
| 198 | 01/23 | SS-23-0000178 | 579 | EMM41.11~ | N/A | . | 93.02 | 100 | . | . | 100 | . | . | . | . | 100 | 100 | . | . |
| 199 | 01/23 | SS-23-0000179 | 433 | EMM49.0 | N/A | . | . | 100 | . | . | 100 | 100 | 100 | . | . | . | . | . | . |
| 200 | 01/23 | SS-23-0000180 | 28 | EMM1.0 | M1UK | 100 | 91.88 | 100 | 100 | 100 | 100 | . | . | 100 | . | . | . | . | 100 |
| 201 | 01/23 | SS-23-0000181 | 36 | EMM12.0 | N/A | . | 95.01 | 100 | . | . | 100 | 100 | 100 | . | . | . | . | 100 | . |
| 202 | 01/23 | SS-23-0000187 | 120 | EMM74.0~ | N/A | . | 100 | 100 | 100 | 100 | 100 | 98.73 | . | . | . | . | . | . | 100 |
| 203 | 01/23 | SS-23-0000189 | 39 | EMM4.0 | N/A | . | 100 | 100 | . | 100 | . | . | . | . | . | . | . | 100 | 100 |
| 204 | 01/23 | SS-23-0000191 | 101 | EMM89.0 | N/A | . | 100 | 100 | . | . | 100 | . | . | . | . | . | . | . | . |
| 205 | 01/23 | SS-23-0000194 | 334 | EMM82.0 | N/A | . | 100 | 100 | . | . | 100 | 100 | 100 | . | . | . | . | . | . |
| 206 | 01/23 | SS-23-0000195 | 36 | EMM12.0 | N/A | . | 100 | 100 | . | 100 | 100 | 100 | 100 | . | . | . | . | . | 100 |
| 207 | 01/23 | SS-23-0000197 | 120 | EMM74.0 | N/A | . | 100 | 100 | 100 | 100 | 100 | 98.73 | . | . | . | . | . | . | 100 |
| 208 | 01/23 | SS-23-0000199 | 36 | EMM12.7 | N/A | . | 100 | 100 | . | 100 | 100 | 100 | 100 | . | . | . | . | . | 100 |
| 209 | 01/23 | SS-23-0000201 | 82 | EMM92.0 | N/A | . | 100 | 100 | . | 100 | 100 | . | . | 100 | . | . | . | . | 100 |
| 210 | 01/23 | SS-23-0000202 | 63 | EMM77.0 | N/A | . | 99.86 | 100 | . | . | . | . | . | . | 97.56 | . | . | . | . |
| 211 | 01/23 | SS-23-0000203 | 172 | EMM59.0 | N/A | . | 100 | 100 | . | 100 | 100 | . | . | . | 100 | . | . | . | 100 |
| 212 | 01/23 | SS-23-0000206 | 36 | EMM12.0 | N/A | . | 100 | 100 | . | 100 | 100 | 100 | 100 | . | . | . | . | . | 100 |
| 213 | 01/23 | SS-23-0000209 | 28 | EMM1.0 | M1UK | 100 | 100 | 100 | 100 | . | 100 | . | . | 100 | . | . | . | . | . |
| 214 | 01/23 | SS-23-0000210 | 433 | EMM49.0 | N/A | . | . | 100 | 100 | . | 100 | 100 | 100 | . | . | . | . | . | . |
| 215 | 01/23 | SS-23-0000212 | 82 | EMM92.0 | N/A | . | 100 | 100 | . | 100 | 100 | . | . | 100 | . | . | . | . | 100 |
| 216 | 01/23 | SS-23-0000218 | 82 | EMM92.0 | N/A | . | 100 | 100 | . | 100 | 100 | . | . | 100 | . | . | . | . | 100 |
| 217 | 01/23 | SS-23-0000225 | 36 | EMM12.0 | N/A | . | 100 | 100 | . | 100 | 100 | 100 | 100 | . | . | . | . | . | 100 |
| 218 | 01/23 | SS-23-0000227 | 28 | EMM1.0 | M1UK | 100 | 100 | 100 | 100 | . | 100 | . | . | 100 | . | . | . | . | . |
| 219 | 01/23 | SS-23-0000233 | 82 | EMM92.0 | N/A | . | 100 | 100 | . | 100 | 100 | . | . | 100 | . | . | . | . | 100 |
| 220 | 02/23 | SS-23-0000236 | 1176 | EMM28.5 | N/A | . | 100 | 100 | . | . | . | . | . | . | . | . | . | . | . |
| 221 | 02/23 | SS-23-0000239 | 247 | EMM68.3 | N/A | . | 100 | 100 | . | 100 | 100 | . | . | 100 | . | 100 | 100 | . | 100 |
| 222 | 02/23 | SS-23-0000242 | 28 | EMM1.3 | M1UK | 100 | 100 | 100 | 100 | . | 100 | . | . | 100 | . | . | . | . | . |
| 223 | 02/23 | SS-23-0000244 | 334 | EMM82.0 | N/A | . | 100 | 100 | . | . | 100 | 100 | 100 | . | . | . | . | . | . |
| 224 | 02/23 | SS-23-0000250 | 334 | EMM82.0 | N/A | . | 100 | 100 | . | . | 100 | 100 | 100 | . | . | . | . | . | . |
| 225 | 02/23 | SS-23-0000255 | 172 | EMM59.0 | N/A | . | 100 | 100 | . | 100 | 100 | . | . | 100 | 100 | . | . | . | 100 |
| 226 | 02/23 | SS-23-0000260 | 579 | EMM41.11 | N/A | . | 100 | 100 | . | 100 | 100 | . | . | . | . | 100 | 100 | . | 100 |
| 227 | 02/23 | SS-23-0000261 | 334 | EMM82.0 | N/A | . | 100 | 100 | . | . | 100 | 100 | 100 | . | . | . | . | . | . |
| 228 | 02/23 | SS-23-0000265 | 433 | EMM49.0 | N/A | . | . | 100 | . | . | 100 | 100 | 100 | . | . | . | . | . | . |
| 229 | 02/23 | SS-23-0000266 | 28 | EMM1.0 | M1UK | 100 | 100 | 100 | 100 | 100 | 100 | . | . | 100 | . | . | . | . | 100 |
| 230 | 02/23 | SS-23-0000270 | 36 | EMM12.40 | N/A | . | 100 | 100 | . | 100 | 100 | 100 | 100 | . | . | . | . | 100 | 100 |
| 231 | 02/23 | SS-23-0000273 | 1176 | EMM28.5 | N/A | . | 100 | 100 | . | . | . | . | . | . | . | . | . | . | . |
| 232 | 02/23 | SS-23-0000274 | 909 | EMM81.0 | N/A | . | . | 100 | . | . | 100 | 100 | . | . | . | . | . | . | . |
| 233 | 02/23 | SS-23-0000276 | 36 | EMM12.7 | N/A | . | 100 | 100 | . | 100 | 100 | 100 | 100 | . | . | . | . | . | 100 |
| 234 | 02/23 | SS-23-0000279 | 433 | EMM49.0 | N/A | . | . | 100 | 100 | . | 100 | 100 | 100 | . | . | . | . | . | . |
| 235 | 02/23 | SS-23-0000281 | 28 | EMM1.0 | M1UK | 100 | 100 | 100 | 100 | . | 100 | . | . | 100 | . | . | . | . | . |
| 236 | 02/23 | SS-23-0000288 | 28 | EMM1.0 | M1global | 100 | 100 | 100 | 100 | . | 100 | . | . | 100 | . | . | . | . | . |
| 237 | 02/23 | SS-23-0000289 | 909 | EMM81.0 | N/A | . | . | 100 | . | . | 100 | 100 | . | . | . | . | . | . | . |
| 238 | 02/23 | SS-23-0000291 | 36 | EMM12.0 | N/A | . | 100 | 100 | . | 100 | 100 | 100 | 100 | . | . | . | . | . | 100 |
| 239 | 02/23 | SS-23-0000297 | 1176 | EMM28.5 | N/A | . | 100 | 100 | . | . | . | . | . | . | . | . | . | . | . |
| 240 | 02/23 | SS-23-0000298 | 82 | EMM92.0 | N/A | . | 100 | 100 | . | 100 | 100 | . | . | 100 | . | . | . | . | 100 |
| 241 | 02/23 | SS-23-0000302 | 28 | EMM1.0 | M1global | 93.2 | 100 | 100 | 100 | . | 100 | . | . | 100 | . | . | . | . | . |
| 242 | 02/23 | SS-23-0000309 | - | EMM74.0 | N/A | . | 100 | 100 | 100 | 100 | 100 | 98.73 | . | . | . | . | . | . | 100 |
| 243 | 02/23 | SS-23-0000312 | 909 | EMM81.0 | N/A | . | . | 100 | . | . | 100 | 100 | . | . | . | . | . | . | . |
| 244 | 02/23 | SS-23-0000315 | 579 | EMM41.11 | N/A | . | 100 | 100 | . | 100 | 100 | . | . | . | . | 100 | 100 | . | 100 |
| 245 | 02/23 | SS-23-0000317 | 433 | EMM49.0 | N/A | . | . | 100 | . | . | 100 | 100 | 100 | . | . | . | . | . | . |
| 246 | 02/23 | SS-23-0000325 | 52 | EMM28.0 | N/A | . | 100 | 100 | . | 100 | 100 | . | . | 100 | . | . | . | . | 100 |
| 247 | 02/23 | SS-23-0000330 | 28 | EMM1.0 | M1global | 93.2 | 100 | 100 | 100 | . | 100 | . | . | 100 | . | . | . | . | . |
| 248 | 02/23 | SS-23-0000331 | 28 | EMM1.0 | M1global | 93.2 | 100 | 100 | 100 | . | 100 | . | . | 100 | . | . | . | . | . |
| 249 | 02/23 | SS-23-0000333 | 334 | EMM82.0 | N/A | . | 100 | 100 | . | . | 100 | 100 | 100 | . | . | . | . | . | . |
| 250 | 02/23 | SS-23-0000336 | 382 | EMM6.4 | N/A | . | 100 | 100 | 100 | . | 100 | 98.73 | . | . | . | . | . | 100 | . |
| 251 | 02/23 | SS-23-0000338 | 36 | EMM12.8 | N/A | . | 100 | 100 | . | 100 | 100 | 100 | 100 | . | . | . | . | . | 100 |
| 252 | 02/23 | SS-23-0000343 | 36 | EMM12.0 | N/A | . | 100 | 100 | . | 100 | 100 | 100 | 100 | . | . | . | . | . | 100 |
| 253 | 02/23 | SS-23-0000344 | 39 | EMM4.0 | N/A | . | 100 | 100 | . | 100 | . | . | . | . | . | . | . | 100 | 100 |
| 254 | 02/23 | SS-23-0000345 | 28 | EMM1.0 | M1intermediate | 100 | . | 100 | . | . | . | . | . | . | . | . | . | . | 100 |
| 255 | 02/23 | SS-23-0000346 | 55 | EMM2.0 | N/A | . | . | 100 | . | 100 | 100 | . | . | . | 100 | . | . | . | 100 |
| 256 | 02/23 | SS-23-0000347 | 28 | EMM1.0 | M1global | 93.2 | 100 | 100 | 100 | . | 100 | . | . | 100 | . | . | . | . | . |
| 257 | 02/23 | SS-23-0000353 | 399 | EMM77.0 | N/A | . | 86.18 | 100 | . | . | 100 | . | . | 100 | . | 100 | 100 | . | . |
| 258 | 02/23 | SS-23-0000354 | 36 | EMM12.8 | N/A | . | 100 | 100 | . | 100 | 100 | 100 | 100 | . | . | . | . | . | 100 |
| 259 | 02/23 | SS-23-0000355 | 36 | EMM12.8 | N/A | . | 100 | 100 | . | 100 | 100 | 100 | 100 | . | . | . | . | . | 100 |
| 260 | 02/23 | SS-23-0000356 | 36 | EMM12.40 | N/A | . | 100 | 100 | . | 100 | 100 | 100 | 100 | . | . | . | . | 100 | 100 |
| 261 | 02/23 | SS-23-0000359 | 120 | EMM74.0 | N/A | . | 100 | 100 | 100 | 100 | 100 | 98.73 | . | . | . | . | . | . | 100 |
| 262 | 02/23 | SS-23-0000360 | 433 | EMM49.0 | N/A | . | . | 100 | 100 | . | 100 | 100 | 100 | . | . | . | . | . | . |
| 263 | 02/23 | SS-23-0000363 | 28 | EMM1.0 | M1UK | 100 | 100 | 100 | 100 | 100 | 100 | . | . | 100 | . | . | . | . | 100 |
| 264 | 02/23 | SS-23-0000366 | 579 | EMM41.11 | N/A | . | 100 | 100 | . | 100 | 100 | . | . | . | . | 100 | 100 | . | 100 |
| 265 | 02/23 | SS-23-0000370 | 36 | EMM12.0 | N/A | . | 100 | 100 | . | 100 | 100 | 100 | 100 | . | . | . | . | . | 100 |
| 266 | 02/23 | SS-23-0000371 | 28 | EMM1.0 | M1global | 93.2 | 100 | 100 | 100 | . | 100 | . | . | 100 | . | . | . | . | . |
| 267 | 02/23 | SS-23-0000375 | 36 | EMM12.8 | N/A | . | 100 | 100 | . | 100 | 100 | 100 | 100 | . | . | . | . | . | 100 |
| 268 | 02/23 | SS-23-0000377 | 172 | EMM59.0 | N/A | . | 100 | 100 | . | 100 | 100 | . | . | 100 | 100 | . | . | . | 100 |
| 269 | 02/23 | SS-23-0000380 | 39 | EMM4.0 | N/A | . | 100 | 100 | . | 100 | . | . | . | . | . | . | . | 100 | 100 |
| 270 | 02/23 | SS-23-0000382 | 28 | EMM1.0 | M1global | 93.2 | 100 | 100 | 100 | . | 100 | . | . | 100 | . | . | . | . | . |
| 271 | 02/23 | SS-23-0000383 | 28 | EMM1.0 | M1UK | 100 | 100 | 100 | 100 | 100 | 100 | . | . | 100 | . | . | . | . | 100 |
| 272 | 02/23 | SS-23-0000384 | 579 | EMM41.11 | N/A | . | 100 | 100 | . | 100 | 100 | . | . | . | . | 100 | 100 | . | 100 |
| 273 | 02/23 | SS-23-0000388 | 36 | EMM12.40 | N/A | . | 100 | 100 | . | 100 | 100 | 100 | 100 | . | . | . | . | 100 | 100 |
| 274 | 02/23 | SS-23-0000389 | 347 | EMM53.0 | N/A | . | 100 | 100 | . | . | 100 | . | . | . | . | . | . | . | . |
| 275 | 02/23 | SS-23-0000393 | 28 | EMM1.3 | M1UK | 100 | 87.04 | 100 | 100 | . | 100 | . | . | 100 | . | . | . | . | . |
| 276 | 02/23 | SS-23-0000394 | 909 | EMM81.0 | N/A | . | . | 100 | . | . | 100 | 100 | . | . | . | . | . | . | . |
| 277 | 02/23 | SS-23-0000400 | 28 | EMM1.0 | M1UK | 100 | 100 | 100 | 100 | 100 | 100 | . | . | 100 | . | . | . | . | 100 |
| 278 | 02/23 | SS-23-0000401 | 28 | EMM1.0 | M1UK | 100 | 100 | 100 | 100 | 100 | 100 | . | . | 100 | . | . | . | . | 100 |
| 279 | 02/23 | SS-23-0000402 | 36 | EMM12.0 | N/A | . | 100 | 100 | . | 100 | 100 | 100 | 100 | . | . | . | . | . | 100 |
| 280 | 02/23 | SS-23-0000403 | 28 | EMM1.0 | M1UK | 100 | 100 | 100 | 100 | 100 | 100 | . | . | 100 | . | . | . | . | 100 |
| 281 | 02/23 | SS-23-0000404 | 120 | EMM74.0 | N/A | . | 100 | 100 | 100 | 100 | 100 | 98.73 | . | . | . | . | . | . | 100 |
| 282 | 02/23 | SS-23-0000405 | 28 | EMM1.0 | M1global | 93.2 | 100 | 100 | 100 | . | 100 | . | . | 100 | . | . | . | . | . |
| 283 | 02/23 | SS-23-0000406 | 36 | EMM12.0 | N/A | . | 100 | 100 | . | 100 | 100 | 100 | 100 | . | . | . | . | . | 100 |
| 284 | 02/23 | SS-23-0000409 | 28 | EMM1.0 | M1global | 96.76 | 100 | 100 | 100 | . | 100 | . | . | 100 | . | . | . | . | . |
| 285 | 02/23 | SS-23-0000481 | 36 | EMM12.8 | N/A | . | 100 | 100 | . | 100 | 100 | 100 | 100 | . | . | . | . | . | 100 |
| 286 | 02/23 | SS-23-0000495 | 382 | EMM6.4 | N/A | . | 100 | 100 | 100 | . | 100 | 98.73 | . | . | . | . | . | 100 | 100 |
| 287 | 02/23 | SS-23-0000412 | 120 | EMM74.0 | N/A | . | 100 | 100 | 100 | 100 | 100 | 98.73 | . | . | . | . | . | . | 100 |
| 288 | 02/23 | SS-23-0000416 | 28 | EMM1.0 | M1UK | 100 | 100 | 100 | 100 | . | 100 | . | . | 100 | . | . | . | . | . |
| 289 | 02/23 | SS-23-0000417 | 28 | EMM1.0 | M1global | 100 | 100 | 100 | 100 | 100 | 100 | . | . | 100 | . | . | . | . | 100 |
| 290 | 02/23 | SS-23-0000421 | 382 | EMM6.4 | N/A | . | 100 | 100 | 100 | . | 100 | 98.73 | . | . | . | . | . | 100 | . |
| 291 | 02/23 | SS-23-0000424 | 82 | EMM92.0 | N/A | . | 100 | 100 | . | 100 | 100 | . | . | 100 | . | . | . | . | 100 |
| 292 | 02/23 | SS-23-0000427 | 28 | EMM1.0 | M1UK | 100 | 100 | 100 | 100 | 100 | 100 | . | . | 100 | . | . | . | . | 100 |
| 293 | 02/23 | SS-23-0000429 | 28 | EMM1.0 | M1global | 93.2 | 100 | 100 | 100 | . | 100 | . | . | 100 | . | . | . | . | . |
| 294 | 02/23 | SS-23-0000430 | 55 | EMM2.0 | N/A | . | . | 100 | . | 100 | 100 | . | . | . | 100 | . | . | . | 100 |
| 295 | 02/23 | SS-23-0000434 | 28 | EMM1.0 | M1global | 93.2 | 100 | 100 | 100 | . | 100 | . | . | 100 | . | . | . | . | . |
| 296 | 02/23 | SS-23-0000436 | 82 | EMM92.0 | N/A | . | 100 | 100 | . | 100 | 100 | . | . | 100 | . | . | . | . | 100 |
| 297 | 02/23 | SS-23-0000437 | 28 | EMM1.0 | M1UK | 100 | 100 | 100 | 100 | 100 | 100 | . | . | 100 | . | . | . | . | 100 |
| 298 | 02/23 | SS-23-0000440 | 433 | EMM49.0 | N/A | . | . | 100 | . | . | 100 | 100 | 100 | . | . | . | . | . | . |
| 299 | 02/23 | SS-23-0000443 | 36 | EMM12.8 | N/A | . | 100 | 100 | . | 100 | 100 | 100 | 100 | . | . | . | . | . | 100 |
| 300 | 02/23 | SS-23-0000444 | 28 | EMM1.0 | M1UK | 100 | 100 | 100 | 100 | 100 | 100 | . | . | 100 | . | . | . | . | 100 |
| 301 | 02/23 | SS-23-0000446 | 28 | EMM1.0 | M1UK | 100 | 100 | 100 | 100 | 100 | 100 | . | . | 100 | . | . | . | . | 100 |
| 302 | 02/23 | SS-23-0000448 | 347 | EMM53.0 | N/A | . | 100 | 100 | . | . | 100 | . | . | . | . | . | . | . | . |
| 303 | 02/23 | SS-23-0000456 | - | EMM74.0 | N/A | . | 100 | 100 | 100 | 100 | 100 | 98.73 | . | . | . | . | . | . | 100 |
| 304 | 03/23 | SS-23-0000449 | 28 | EMM1.3 | M1UK | . | 100 | 100 | 100 | . | 100 | . | . | 100 | . | . | . | . | . |
| 305 | 03/23 | SS-23-0000455 | 55 | EMM2.0 | N/A | . | . | 100 | . | 100 | 100 | . | . | . | 100 | . | . | . | 100 |
| 306 | 03/23 | SS-23-0000457 | 101 | EMM89.0 | N/A | . | 100 | 100 | . | 100 | 100 | . | . | . | . | . | . | . | 100 |
| 307 | 03/23 | SS-23-0000458 | 28 | EMM1.3 | M1UK | . | 100 | 100 | 100 | . | 100 | . | . | 100 | . | . | . | . | . |
| 308 | 03/23 | SS-23-0000459 | 55 | EMM2.0 | N/A | . | . | 100 | . | 100 | 100 | . | . | . | 100 | . | . | . | 100 |
| 309 | 03/23 | SS-23-0000460 | 347 | EMM53.0 | N/A | . | 100 | 100 | . | . | 100 | . | . | . | . | . | . | . | . |
| 310 | 03/23 | SS-23-0000461 | 36 | EMM12.0 | N/A | . | 100 | 100 | . | 100 | 100 | 100 | 100 | . | . | . | . | . | 100 |
| 311 | 03/23 | SS-23-0000465 | 28 | EMM1.0 | M1UK | 100 | 100 | 100 | 100 | 100 | 100 | . | . | 100 | . | . | . | . | 100 |
| 312 | 03/23 | SS-23-0000466 | 347 | EMM53.0 | N/A | . | 100 | 100 | . | . | 100 | . | . | . | . | . | . | . | . |
| 313 | 03/23 | SS-23-0000467 | 36 | EMM12.8 | N/A | . | 100 | 100 | . | 100 | 100 | 100 | 100 | . | . | . | . | . | 100 |
| 314 | 03/23 | SS-23-0000473 | 28 | EMM1.3 | M1UK | 100 | 100 | 100 | 100 | . | 100 | . | . | 100 | . | . | . | . | . |
| 315 | 03/23 | SS-23-0000474 | 334 | EMM82.0 | N/A | . | 100 | 100 | . | . | 100 | 100 | 100 | . | . | . | . | . | . |
| 316 | 03/23 | SS-23-0000475 | 347 | EMM53.0 | N/A | . | 100 | 100 | . | . | 100 | . | . | . | . | . | . | . | . |
| 317 | 03/23 | SS-23-0000482 | 82 | EMM92.0 | N/A | . | 100 | 100 | . | 100 | 100 | . | . | 100 | . | . | . | . | 100 |
| 318 | 03/23 | SS-23-0000489 | 28 | EMM1.74 | M1UK | 100 | 100 | 100 | 100 | . | 100 | . | . | 100 | . | . | . | . | . |
| 319 | 03/23 | SS-23-0000493 | 28 | EMM1.0 | M1UK | 100 | 100 | 100 | 100 | . | 100 | . | . | 100 | . | . | . | . | . |
| 320 | 03/23 | SS-23-0000494 | 28 | EMM1.0 | M1UK | 100 | 100 | 100 | 100 | 100 | 100 | . | . | 100 | . | . | . | . | 100 |
| 321 | 03/23 | SS-23-0000496 | 347 | EMM53.0 | N/A | . | 100 | 100 | . | . | 100 | . | . | . | . | . | . | . | . |
| 322 | 03/23 | SS-23-0000500 | 36 | EMM12.8 | N/A | . | 100 | 100 | . | 100 | 100 | 100 | 100 | . | . | . | . | . | 100 |
| 323 | 03/23 | SS-23-0000502 | 36 | EMM12.8 | N/A | . | 100 | 100 | . | 100 | 100 | 100 | 100 | . | . | . | . | . | 100 |
| 324 | 03/23 | SS-23-0000507 | 334 | EMM82.0 | N/A | . | 100 | 100 | . | . | 100 | 100 | 96.9 | . | . | . | . | . | . |
| 325 | 03/23 | SS-23-0000508 | 36 | EMM12.8 | N/A | . | 100 | 100 | . | 100 | 100 | 100 | 100 | . | . | . | . | . | 100 |
| 326 | 03/23 | SS-23-0000511 | 28 | EMM1.0 | M1global | 93.2 | 100 | 100 | 100 | . | 100 | . | . | 100 | . | . | . | . | . |
| 327 | 03/23 | SS-23-0000512 | 28 | EMM1.0 | M1UK | 100 | 100 | 100 | 100 | 100 | 100 | . | . | 100 | . | . | . | . | 100 |
| 328 | 03/23 | SS-23-0000516 | 28 | EMM1.0 | M1UK | 100 | 100 | 100 | 100 | 100 | 100 | . | . | 100 | . | . | . | . | 100 |
| 329 | 03/23 | SS-23-0000518 | 82 | EMM92.0 | N/A | . | 100 | 100 | . | 100 | 100 | . | . | 100 | . | . | . | . | 100 |
| 330 | 03/23 | SS-23-0000522 | 433 | EMM49.0 | N/A | . | . | 100 | 100 | . | 100 | 100 | 100 | . | . | . | . | . | . |
| 331 | 03/23 | SS-23-0000525 | 36 | EMM12.0 | N/A | . | 100 | 100 | . | . | 100 | 100 | 100 | . | . | . | . | 100 | . |
| 332 | 03/23 | SS-23-0000533 | 909 | EMM81.0 | N/A | . | . | 100 | . | . | 100 | 100 | . | . | . | . | . | . | . |
| 333 | 03/23 | SS-23-0000534 | 334 | EMM82.0 | N/A | . | 100 | 100 | . | . | 100 | 100 | 100 | . | . | . | . | . | . |
| 334 | 03/23 | SS-23-0000535 | 28 | EMM1.0 | M1UK | 100 | 100 | 100 | 100 | 100 | 100 | . | . | 100 | . | . | . | . | 100 |
| 335 | 03/23 | SS-23-0000538 | 579 | EMM41.11 | N/A | . | 100 | 100 | . | 100 | 100 | . | . | . | . | 100 | 100 | . | 100 |
| 336 | 03/23 | SS-23-0000539 | 347 | EMM53.0 | N/A | . | 100 | 100 | . | . | 100 | . | . | . | . | . | . | . | . |
| 337 | 03/23 | SS-23-0000541 | 82 | EMM92.0 | N/A | . | 100 | 100 | . | 100 | 100 | . | . | 100 | . | . | . | . | 100 |
| 338 | 03/23 | SS-23-0000544 | 28 | EMM1.0 | M1global | 93.2 | 100 | 100 | 100 | . | 100 | . | . | 100 | . | . | . | . | . |
| 339 | 03/23 | SS-23-0000547 | 36 | EMM12.8 | N/A | . | 100 | 100 | . | 100 | 100 | 100 | 100 | . | . | . | . | . | 100 |
| 340 | 03/23 | SS-23-0000552 | 853 | EMM83.1 | N/A | . | 100 | 100 | . | . | 100 | . | . | . | . | . | . | . | . |
| 341 | 03/23 | SS-23-0000554 | 39 | EMM4.0 | N/A | . | 100 | 100 | . | 100 | . | . | . | . | . | . | . | 100 | 100 |
| 342 | 03/23 | SS-23-0000557 | 36 | EMM12.8 | N/A | . | 100 | 100 | . | 100 | 100 | 100 | 100 | . | . | . | . | . | 100 |
| 343 | 03/23 | SS-23-0000560 | 28 | EMM1.3 | M1UK | 100 | 95.58 | 100 | 100 | . | 100 | . | . | . | . | . | . | . | . |
| 344 | 03/23 | SS-23-0000563 | 458 | EMM28.0 | N/A | . | 100 | 100 | . | 100 | 100 | . | . | 100 | . | . | . | . | 100 |
| 345 | 03/23 | SS-23-0000565 | 172 | EMM59.0 | N/A | . | 95.01 | 100 | . | 100 | 100 | . | . | 100 | 100 | . | . | . | 100 |
| 346 | 03/23 | SS-23-0000567 | - | EMM74.0 | N/A | . | 93.02 | 100 | 100 | 100 | 100 | 98.73 | . | . | . | . | . | . | 100 |
| 347 | 03/23 | SS-23-0000568 | 172 | EMM59.0 | N/A | . | 100 | 100 | . | 100 | 100 | . | . | 100 | 100 | . | . | . | 100 |
| 348 | 03/23 | SS-23-0000569 | 28 | EMM1.3 | M1UK | 100 | 100 | 100 | 100 | . | 100 | . | . | 100 | . | . | . | . | . |
| 349 | 03/23 | SS-23-0000570 | 334 | EMM82.0 | N/A | . | 100 | 100 | . | . | 100 | 100 | 100 | . | . | . | . | . | . |
| 350 | 03/23 | SS-23-0000571 | 458 | EMM28.0 | N/A | . | 100 | 100 | . | 100 | 100 | . | . | 100 | . | . | . | . | 100 |
| 351 | 03/23 | SS-23-0000574 | 172 | EMM59.0 | N/A | . | 91.74 | 100 | . | 100 | 100 | . | . | 100 | 100 | . | . | . | 100 |
| 352 | 03/23 | SS-23-0000575 | 28 | EMM1.3 | M1UK | 100 | 100 | 100 | 100 | . | 100 | . | . | 100 | . | . | . | . | . |
| 353 | 03/23 | SS-23-0000582 | 36 | EMM12.8 | N/A | . | . | 100 | . | 100 | 100 | 100 | 100 | . | . | . | . | . | 100 |
| 354 | 03/23 | SS-23-0000584 | 36 | EMM12.8 | N/A | . | 100 | 100 | . | 100 | 100 | 100 | 100 | . | . | . | . | . | 100 |
| 355 | 03/23 | SS-23-0000590 | 50 | EMM76.0 | N/A | . | 94.3 | 100 | . | . | 100 | . | . | 100 | . | 100 | 100 | . | . |
| 356 | 03/23 | SS-23-0000592 | 579 | EMM41.11 | N/A | . | 100 | 100 | . | 100 | 100 | . | . | . | . | 100 | 100 | . | 100 |
| 357 | 03/23 | SS-23-0000593 | 46 | EMM22.0 | N/A | . | 88.18 | 100 | 100 | . | 100 | . | . | . | . | . | . | 100 | . |
| 358 | 03/23 | SS-23-0000594 | 36 | EMM12.8 | N/A | . | 100 | 100 | . | 100 | 100 | 100 | 100 | . | . | . | . | . | 100 |
| 359 | 03/23 | SS-23-0000596 | 28 | EMM1.0 | M1global | . | 100 | 100 | 100 | . | 100 | . | . | 100 | . | . | . | . | . |
| 360 | 03/23 | SS-23-0000599 | 120 | EMM74.0 | N/A | . | 100 | 100 | 100 | 100 | 100 | . | . | . | . | . | . | . | 100 |
| 361 | 03/23 | SS-23-0000606 | 28 | EMM1.0 | M1UK | 100 | 100 | 100 | 100 | 100 | 100 | . | . | 100 | . | . | . | . | 100 |
| 362 | 03/23 | SS-23-0000609 | 28 | EMM1.0 | M1UK | 100 | 100 | 100 | 100 | . | 100 | . | . | 100 | . | . | . | . | . |
| 363 | 03/23 | SS-23-0000616 | 28 | EMM1.0 | M1UK | 100 | 100 | 100 | 100 | . | 100 | . | . | 100 | . | . | . | . | . |
| 364 | 03/23 | SS-23-0000617 | 28 | EMM1.0 | M1global | 93.2 | 91.88 | 100 | 100 | . | 100 | . | . | 100 | . | . | . | . | . |
| 365 | 03/23 | SS-23-0000618 | 28 | EMM1.0 | M1UK | 94.82 | 100 | 100 | 100 | 100 | 100 | . | . | 100 | . | . | . | . | 100 |
| 366 | 03/23 | SS-23-0000620 | 579 | EMM41.11 | N/A | . | 100 | 100 | . | 100 | 100 | . | . | . | . | 100 | 100 | . | 100 |
| 367 | 03/23 | SS-23-0000621 | 433 | EMM49.0 | N/A | . | . | 100 | . | . | 100 | 100 | 100 | . | . | . | . | . | . |
| 368 | 03/23 | SS-23-0000622 | 28 | EMM1.0 | M1UK | 100 | 100 | 100 | 100 | 100 | 100 | . | . | 100 | . | . | . | . | 100 |
| 369 | 03/23 | SS-23-0000624 | 120 | EMM74.0 | N/A | . | 100 | 100 | 100 | 100 | 100 | 98.73 | . | . | . | . | . | . | 100 |
| 370 | 03/23 | SS-23-0000627 | 36 | EMM12.8 | N/A | . | 100 | 100 | . | 100 | 100 | 100 | 100 | . | . | . | . | . | 100 |
| 371 | 03/23 | SS-23-0000633 | 120 | EMM74.0 | N/A | . | 100 | 100 | 100 | 100 | 100 | 98.73 | . | . | . | . | . | . | 100 |
| 372 | 03/23 | SS-23-0000635 | 50 | EMM76.0 | N/A | . | 100 | 100 | . | . | 100 | . | . | . | . | 100 | 100 | . | . |
| 373 | 03/23 | SS-23-0000662 | 82 | EMM92.0 | N/A | . | 100 | 100 | . | 100 | 100 | . | . | 100 | . | . | . | . | 100 |
| 374 | 04/23 | SS-23-0000642 | 36 | EMM12.8 | N/A | . | 100 | 100 | . | 100 | 100 | 100 | 100 | . | . | . | . | . | 100 |
| 375 | 04/23 | SS-23-0000645 | 579 | EMM41.11 | N/A | . | 100 | 100 | . | 100 | 100 | . | . | . | . | 100 | 100 | . | 100 |
| 376 | 04/23 | SS-23-0000649 | 120 | EMM74.0~ | N/A | . | 100 | 100 | 100 | 100 | 100 | 98.73 | . | . | . | . | . | . | 100 |
| 377 | 04/23 | SS-23-0000650 | 28 | EMM1.0 | M1global | 93.2 | 100 | 100 | 100 | . | 100 | . | . | 100 | . | . | . | . | . |
| 378 | 04/23 | SS-23-0000652 | 46 | EMM22.0 | N/A | . | 100 | 100 | . | . | 100 | . | . | . | . | . | . | 100 | . |
| 379 | 04/23 | SS-23-0000654 | 36 | EMM12.8 | N/A | . | 100 | 100 | . | 100 | 100 | 100 | 100 | . | . | . | . | . | 100 |
| 380 | 04/23 | SS-23-0000655 | 853 | EMM83.1 | N/A | . | 91.88 | 100 | . | . | 100 | . | . | . | . | . | . | . | . |
| 381 | 04/23 | SS-23-0000659 | 28 | EMM1.0 | M1global | 93.2 | 100 | 100 | 100 | . | 100 | . | . | 100 | . | . | . | . | . |
| 382 | 04/23 | SS-23-0000660 | 433 | EMM49.0 | N/A | . | . | 100 | 100 | . | 100 | 100 | 100 | . | . | . | . | . | . |
| 383 | 04/23 | SS-23-0000663 | 36 | EMM12.8 | N/A | . | 100 | 100 | . | 100 | 100 | 100 | 100 | . | . | . | . | . | 100 |
| 384 | 04/23 | SS-23-0000667 | 28 | EMM1.0 | M1global | 93.2 | 100 | 100 | 100 | . | 100 | . | . | 100 | . | . | . | . | . |
| 385 | 04/23 | SS-23-0000673 | 347 | EMM53.0 | N/A | . | 100 | 100 | . | . | 100 | . | . | . | . | . | . | . | . |
| 386 | 04/23 | SS-23-0000676 | 12 | EMM91.0 | N/A | . | 100 | 100 | . | . | 100 | . | . | . | . | . | . | 100 | . |
| 387 | 04/23 | SS-23-0000677 | 36 | EMM12.8 | N/A | . | 87.04 | 100 | . | 100 | . | 100 | 100 | . | . | . | . | . | 95.78 |
| 388 | 04/23 | SS-23-0000679 | 172 | EMM59.0 | N/A | . | 100 | 100 | . | 100 | 100 | . | . | 100 | 100 | . | . | . | 100 |
| 389 | 04/23 | SS-23-0000682 | 28 | EMM1.0 | M1global | 93.2 | 100 | 100 | 100 | . | 100 | . | . | 100 | . | . | . | . | . |
| 390 | 04/23 | SS-23-0000683 | 334 | EMM82.0 | N/A | . | 100 | 100 | . | . | 100 | 100 | 100 | . | . | . | . | . | . |
| 391 | 04/23 | SS-23-0000684 | 334 | EMM82.0 | N/A | . | 100 | 100 | . | . | 100 | 100 | 100 | . | . | . | . | . | . |
| 392 | 04/23 | SS-23-0000685 | 36 | EMM12.8 | N/A | . | 100 | 100 | . | 100 | 100 | 100 | 100 | . | . | . | . | . | 100 |
| 393 | 04/23 | SS-23-0000687 | 347 | EMM53.0 | N/A | . | 100 | 100 | . | . | 100 | . | . | . | . | . | . | . | . |
| 394 | 04/23 | SS-23-0000689 | 172 | EMM59.0 | N/A | . | 100 | 100 | . | 100 | 100 | . | . | 100 | 100 | . | . | . | 100 |
| 395 | 04/23 | SS-23-0000691 | 347 | EMM53.0 | N/A | . | 100 | 100 | . | . | 100 | . | . | . | . | . | . | . | . |
| 396 | 04/23 | SS-23-0000694 | 433 | EMM49.0 | N/A | . | . | 100 | 100 | . | 100 | 100 | 100 | . | . | . | . | . | . |
| 397 | 04/23 | SS-23-0000697 | 28 | EMM1.3 | M1UK | 90.61 | 100 | 100 | 100 | . | 100 | . | . | 100 | . | . | . | . | . |
| 398 | 04/23 | SS-23-0000698 | 28 | EMM1.0 | M1UK | 100 | 100 | 100 | 100 | 100 | 100 | . | . | 100 | . | . | . | . | 100 |
| 399 | 04/23 | SS-23-0000700 | 579 | EMM41.11 | N/A | . | 100 | 100 | . | 100 | 100 | . | . | . | . | 100 | 100 | . | 100 |
| 400 | 04/23 | SS-23-0000701 | 36 | EMM12.0 | N/A | . | 100 | 100 | . | . | 100 | 100 | 100 | . | . | . | . | 100 | . |
| 401 | 04/23 | SS-23-0000704 | 28 | EMM1.0 | M1UK | 100 | 100 | 100 | 100 | 100 | 100 | . | . | 100 | . | . | . | . | 100 |
| 402 | 04/23 | SS-23-0000711 | 28 | EMM1.0 | M1UK | 100 | 100 | 100 | 100 | 100 | 100 | . | . | 100 | . | . | . | . | 100 |
| 403 | 04/23 | SS-23-0000715 | 28 | EMM1.0 | M1global | 93.2 | 100 | 100 | 100 | . | 100 | . | . | 100 | . | . | . | . | . |
| 404 | 04/23 | SS-23-0000720 | 101 | EMM89.0 | N/A | . | 100 | 100 | . | 100 | 100 | . | . | . | . | . | . | . | 100 |
| 405 | 04/23 | SS-23-0000721 | 28 | EMM1.0 | M1global | 93.2 | 100 | 100 | 100 | . | 100 | . | . | 100 | . | . | . | . | . |
| 406 | 04/23 | SS-23-0000722 | 515 | EMM110.0 | N/A | . | 100 | 100 | . | . | 100 | 100 | . | 100 | . | . | . | . | . |
| 407 | 04/23 | SS-23-0000725 | 458 | EMM28.5~ | N/A | . | 100 | 100 | . | 100 | 100 | . | . | 100 | . | . | . | . | 100 |
| 408 | 04/23 | SS-23-0000726 | 36 | EMM12.7 | N/A | . | 100 | 100 | . | 100 | 100 | 100 | 100 | . | . | . | . | . | 100 |
| 409 | 04/23 | SS-23-0000731 | 120 | EMM74.0 | N/A | . | 100 | 100 | 100 | 100 | 100 | 98.73 | . | . | . | . | . | . | 100 |
| 410 | 04/23 | SS-23-0000733 | 50 | EMM76.0 | N/A | 88.89 | 100 | 100 | . | . | 100 | . | . | 100 | . | 100 | 100 | . | . |
| 411 | 04/23 | SS-23-0000734 | 36 | EMM12.0 | N/A | . | 100 | 100 | . | 100 | 100 | 100 | 100 | . | . | . | . | . | 100 |
| 412 | 04/23 | SS-23-0000739 | 334 | EMM82.0 | N/A | . | 100 | 100 | . | . | 100 | 100 | 100 | . | . | . | . | . | . |
| 413 | 04/23 | SS-23-0000745 | 28 | EMM1.0 | M1global | 93.2 | 100 | 100 | 100 | . | 100 | . | . | 100 | . | . | . | . | . |
| 414 | 04/23 | SS-23-0000746 | 28 | EMM1.0 | M1UK | 100 | 100 | 100 | 100 | 100 | 100 | . | . | 100 | . | . | . | . | 100 |
| 415 | 04/23 | SS-23-0000751 | 1176 | EMM28.5 | N/A | . | 100 | 100 | . | . | . | . | . | . | . | . | . | . | . |
| 416 | 04/23 | SS-23-0000753 | 899 | EMM1.29 | M1global | 98.71 | 100 | 100 | 100 | . | 100 | . | . | 100 | . | . | . | . | . |
| 417 | 04/23 | SS-23-0000754 | 28 | EMM1.0 | M1UK | 100 | 100 | 100 | 100 | 100 | 100 | . | . | 100 | . | . | . | . | 100 |
| 418 | 04/23 | SS-23-0000760 | 28 | EMM1.0 | M1UK | 100 | 100 | 100 | 100 | 100 | 100 | . | . | 100 | . | . | . | . | 100 |
| 419 | 04/23 | SS-23-0000761 | 36 | EMM12.8 | N/A | . | 100 | 100 | . | 100 | 100 | 100 | 100 | . | . | . | . | . | 100 |
| 420 | 04/23 | SS-23-0000762 | 28 | EMM1.0 | M1global | 93.2 | 100 | 100 | 100 | . | 100 | . | . | 100 | . | . | . | . | . |
| 421 | 04/23 | SS-23-0000763 | 28 | EMM1.0 | M1UK | 100 | 100 | 100 | 100 | 100 | 100 | . | . | 100 | . | . | . | . | 100 |
| 422 | 04/23 | SS-23-0000765 | 36 | EMM12.8 | N/A | . | 100 | 100 | . | 100 | 100 | 100 | 100 | . | . | . | . | . | 100 |
| 423 | 04/23 | SS-23-0000767 | 403 | EMM11.0 | N/A | . | 100 | 100 | 100 | 100 | 100 | . | . | . | . | . | . | . | 100 |
| 424 | 04/23 | SS-23-0000769 | 347 | EMM53.0 | N/A | . | 100 | 100 | . | . | 100 | . | . | . | . | . | . | . | . |
| 425 | 04/23 | SS-23-0000771 | 36 | EMM12.0 | N/A | . | 100 | 100 | . | 100 | 100 | 100 | 100 | . | . | . | . | . | 100 |
| 426 | 04/23 | SS-23-0000772 | 36 | EMM12.8 | N/A | . | 100 | 100 | . | 100 | 100 | 100 | 100 | . | . | . | . | . | 100 |
| 427 | 04/23 | SS-23-0000774 | 12 | EMM91.0 | N/A | . | 100 | 100 | . | . | 100 | . | . | . | . | . | . | 100 | . |
| 428 | 04/23 | SS-23-0000778 | 82 | EMM92.0 | N/A | . | 100 | 100 | . | . | 100 | . | . | 100 | . | 100 | 100 | . | . |
| 429 | 04/23 | SS-23-0000779 | 36 | EMM12.8 | N/A | . | 100 | 100 | . | 100 | 100 | 100 | 100 | . | . | . | . | . | 100 |
| 430 | 04/23 | SS-23-0000784 | 36 | EMM12.0 | N/A | . | 100 | 100 | . | 100 | 100 | 100 | 100 | . | . | . | . | . | 100 |
| 431 | 04/23 | SS-23-0000785 | 36 | EMM12.0 | N/A | . | 100 | 100 | . | 100 | 100 | . | . | . | . | . | . | . | 100 |
| 432 | 04/23 | SS-23-0000786 | 172 | EMM59.0 | N/A | . | 100 | 100 | . | 100 | 100 | . | . | 100 | 100 | . | . | . | 100 |
| 433 | 04/23 | SS-23-0000792 | 433 | EMM49.0 | N/A | . | . | 100 | 100 | . | 100 | 100 | 100 | . | . | . | . | . | . |
| 434 | 04/23 | SS-23-0000793 | 36 | EMM12.0 | N/A | . | 100 | 100 | . | 100 | 100 | 100 | 100 | . | . | . | . | . | 100 |
| 435 | 04/23 | SS-23-0000794 | 36 | EMM12.8 | N/A | . | 100 | 100 | . | 100 | 100 | 100 | 100 | . | . | . | . | . | 100 |
| 436 | 04/23 | SS-23-0000797 | 12 | EMM91.0 | N/A | . | 100 | 100 | . | . | 100 | . | . | . | . | . | . | 100 | . |
| 437 | 04/23 | SS-23-0000800 | 579 | EMM41.11 | N/A | . | 100 | 100 | . | 100 | 100 | . | . | . | . | 100 | 100 | . | 100 |
| 438 | 04/23 | SS-23-0000801 | 120 | EMM74.0 | N/A | . | 100 | 100 | 100 | 100 | 100 | 98.73 | . | . | . | . | . | . | 100 |
| 439 | 04/23 | SS-23-0000803 | 36 | EMM12.0 | N/A | . | 100 | 100 | . | . | 100 | 100 | 100 | . | . | . | . | 100 | . |
| 440 | 04/23 | SS-23-0000805 | 382 | EMM6.64 | N/A | . | 94.3 | 100 | 100 | 100 | 100 | 98.73 | . | . | 100 | . | . | . | 100 |
| 441 | 04/23 | SS-23-0000806 | 1176 | EMM28.5 | N/A | . | 100 | 100 | . | . | . | . | . | . | . | . | . | . | . |
| 442 | 04/23 | SS-23-0000807 | 458 | EMM28.0 | N/A | . | 100 | 100 | . | 100 | 100 | . | . | 100 | . | . | . | . | 100 |
| 443 | 04/23 | SS-23-0000811 | 347 | EMM53.0 | N/A | . | 100 | 100 | . | . | 100 | . | . | . | . | . | . | . | . |
| 444 | 04/23 | SS-23-0000814 | 101 | EMM89.0 | N/A | . | 100 | 100 | . | 100 | 100 | . | . | . | . | . | . | . | 100 |
| 445 | 04/23 | SS-23-0000818 | 82 | EMM92.0 | N/A | . | 100 | 100 | . | 100 | 100 | . | . | 100 | . | . | . | . | 100 |
| 446 | 04/23 | SS-23-0000820 | 36 | EMM12.7 | N/A | . | 100 | 100 | . | 100 | 100 | 100 | 100 | . | . | . | . | . | 100 |
| 447 | 04/23 | SS-23-0000821 | 403 | EMM11.0 | N/A | . | 100 | 100 | 100 | 100 | 100 | . | . | . | . | . | . | . | 100 |
| 448 | 04/23 | SS-23-0000825 | 101 | EMM89.0 | N/A | . | 100 | 100 | . | . | 100 | . | . | . | . | . | . | . | . |
| 449 | 04/23 | SS-23-0000827 | 36 | EMM12.7 | N/A | . | 100 | 100 | . | 100 | 100 | 100 | 100 | . | . | . | . | . | 100 |
| 450 | 04/23 | SS-23-0000831 | 82 | EMM92.0 | N/A | . | 100 | 100 | . | 100 | 100 | . | . | 100 | . | . | . | . | 100 |
| 451 | 04/23 | SS-23-0000832 | 62 | EMM87.0 | N/A | . | 100 | 100 | . | . | 100 | . | . | 100 | 97.56 | . | . | . | . |
| 452 | 04/23 | SS-23-0000835 | 36 | EMM12.0 | N/A | . | 100 | 100 | . | 100 | 100 | 100 | 100 | . | . | . | . | . | 100 |
| 453 | 04/23 | SS-23-0000836 | 579 | EMM41.11~ | N/A | . | 100 | 100 | . | 100 | 100 | . | . | . | . | 100 | 100 | . | 100 |
| 454 | 04/23 | SS-23-0000839 | 28 | EMM1.0 | M1global | . | 100 | 100 | 100 | . | 100 | . | . | 100 | . | . | . | . | . |
| 455 | 04/23 | SS-23-0000840 | 120 | EMM74.0 | N/A | . | 100 | 100 | 100 | 100 | 100 | 98.73 | . | . | . | . | . | . | 100 |
| 456 | 04/23 | SS-23-0000847 | 433 | EMM49.0 | N/A | . | . | 100 | 100 | . | 100 | 100 | 100 | . | . | . | . | . | . |
| 457 | 04/23 | SS-23-0000848 | 28 | EMM1.0 | M1global | 93.2 | 100 | 100 | 100 | . | 100 | . | . | 100 | . | . | . | . | . |
| 458 | 04/23 | SS-23-0000849 | 28 | EMM1.0 | M1UK | 100 | 100 | 100 | 100 | 100 | 100 | . | . | 100 | . | . | . | . | 100 |
| 459 | 04/23 | SS-23-0000850 | 36 | EMM12.0 | N/A | . | 100 | 100 | . | 100 | 100 | 100 | 100 | . | . | . | . | . | 100 |
| 460 | 04/23 | SS-23-0000851 | 150 | EMM75.0 | N/A | . | 99.57 | 100 | . | . | 100 | . | . | . | . | 100 | 100 | . | . |
| 461 | 04/23 | SS-23-0000855 | 334 | EMM82.0 | N/A | . | 100 | 100 | . | . | 100 | 100 | 100 | . | . | . | . | . | . |
| 462 | 04/23 | SS-23-0000856 | 28 | EMM1.0 | M1UK | 100 | 100 | 100 | 100 | . | 100 | . | . | 100 | . | . | . | . | . |
| 463 | 04/23 | SS-23-0000860 | 55 | EMM2.0 | N/A | . | . | 100 | . | 100 | 100 | . | . | . | 100 | . | . | . | 100 |
| 464 | 04/23 | SS-23-0000861 | 46 | EMM22.0 | N/A | . | 100 | 100 | 100 | . | 100 | . | . | . | . | . | . | 100 | . |
| 465 | 04/23 | SS-23-0000862 | 334 | EMM82.0 | N/A | . | 100 | 100 | . | . | 100 | 100 | 100 | . | . | . | . | . | . |
| 466 | 04/23 | SS-23-0000863 | 82 | EMM92.0 | N/A | . | 100 | 100 | . | 100 | 100 | . | . | 100 | . | . | . | . | 100 |
| 467 | 04/23 | SS-23-0000865 | 12 | EMM91.0 | N/A | . | 100 | 100 | . | . | 100 | . | . | . | . | . | . | 100 | . |
| 468 | 04/23 | SS-23-0000866 | 334 | EMM82.0 | N/A | . | 100 | 100 | . | . | 100 | 100 | 100 | . | . | . | . | . | . |
| 469 | 04/23 | SS-23-0000871 | 120 | EMM74.0 | N/A | . | 100 | 100 | 100 | 100 | 100 | 98.73 | . | . | . | . | . | . | 100 |
| 470 | 04/23 | SS-23-0000873 | 28 | EMM1.0 | M1UK | 90.61 | 100 | 100 | 100 | . | 100 | . | . | 100 | . | . | . | . | . |
| 471 | 04/23 | SS-23-0000874 | 837 | EMM81.0 | N/A | . | . | 100 | . | . | 100 | 100 | . | . | . | 100 | 100 | . | . |
| 472 | 05/23 | SS-23-0000879 | 347 | EMM53.0 | N/A | . | 100 | 100 | . | . | 100 | . | . | . | . | . | . | . | . |
| 473 | 05/23 | SS-23-0000880 | 28 | EMM1.3 | M1UK | 100 | 100 | 100 | 100 | . | 100 | . | . | 100 | . | . | . | . | . |
| 474 | 05/23 | SS-23-0000883 | 36 | EMM12.8 | N/A | . | 100 | 100 | . | 100 | 100 | 100 | 100 | . | . | . | . | . | 100 |
| 475 | 05/23 | 23PN-129A00025 | 28 | EMM1.0 | M1global | 93.2 | 100 | 100 | 100 | . | 100 | . | . | 100 | . | . | . | . | . |
| 476 | 05/23 | 23PN-136A00021 | 28 | EMM1.0 | M1global | 93.2 | 100 | 100 | 100 | . | 100 | . | . | 100 | . | . | . | . | . |
| 477 | 05/23 | 23PN-136A00028 | 101 | EMM89.0 | N/A | . | 100 | 100 | . | 100 | 100 | . | . | . | . | . | . | . | 100 |
| 478 | 05/23 | 23PN-136A00030 | 28 | EMM1.0 | M1UK | 100 | 100 | 100 | 100 | 100 | 100 | . | . | 100 | . | . | . | . | 100 |
| 479 | 05/23 | 23PN-136A00031 | 28 | EMM1.0 | M1UK | 100 | 100 | 100 | 100 | 100 | 100 | . | . | 100 | . | . | . | . | 100 |
| 480 | 05/23 | 23PN-136A00035 | 36 | EMM12.8 | N/A | . | 100 | 100 | . | 100 | 100 | 100 | 100 | . | . | . | . | . | 100 |
| 481 | 05/23 | 23PN-136A00037 | 347 | EMM53.0 | N/A | . | 100 | 100 | . | . | 100 | . | . | . | . | . | . | . | . |
| 482 | 05/23 | 23PN-136A00039 | 579 | EMM41.11 | N/A | . | 100 | 100 | . | 100 | 100 | . | . | . | . | 100 | 100 | . | 100 |
| 483 | 05/23 | 23PN-136A00045 | 63 | EMM77.0 | N/A | . | 99.86 | 100 | . | . | . | . | . | . | 97.56 | . | . | . | . |
| 484 | 05/23 | 23PN-136A00046 | 101 | EMM89.0 | N/A | . | 100 | 100 | . | 100 | 100 | . | . | . | . | . | . | . | 100 |
| 485 | 05/23 | 23PN-136A00048 | 347 | EMM53.0 | N/A | . | 100 | 100 | . | . | 100 | . | . | . | . | . | . | . | . |
| 486 | 05/23 | 23PN-136A00049 | 28 | EMM1.0 | M1UK | 94.82 | 100 | 100 | 100 | . | 100 | . | . | 100 | . | . | . | . | . |
| 487 | 05/23 | 23PN-136A00050 | 150 | EMM75.0 | N/A | . | 99.57 | 100 | . | . | 100 | . | . | . | . | 100 | 100 | . | . |
| 488 | 05/23 | 23PN-136A00051 | 28 | EMM1.0 | M1UK | 100 | 100 | 100 | 100 | 100 | 100 | . | . | 100 | . | . | . | . | 100 |
| 489 | 05/23 | 23PN-136A00059 | 120 | EMM74.0 | N/A | . | 100 | 100 | 100 | 100 | 100 | 98.73 | . | . | . | . | . | . | 100 |
| 490 | 05/23 | 23PN-136A00060 | 39 | EMM4.0 | N/A | . | 100 | 100 | . | 100 | . | . | . | . | . | . | . | 100 | 100 |
| 491 | 05/23 | 23PN-136A00061 | 172 | EMM59.0 | N/A | . | 100 | 100 | . | 100 | 100 | . | . | 100 | 100 | . | . | . | 100 |
| 492 | 05/23 | 23PN-136A00064 | 433 | EMM49.0 | N/A | . | . | 100 | . | . | 100 | 100 | 100 | . | . | . | . | . | . |
| 493 | 05/23 | 23PN-136A00065 | 46 | EMM22.0 | N/A | . | 100 | 100 | 100 | . | 100 | . | . | . | 100 | . | . | 100 | . |
| 494 | 05/23 | 23PN-136A00066 | - | EMM166.0 | N/A | . | 98.72 | 100 | . | . | 100 | . | . | . | 97.56 | . | . | . | . |
| 495 | 05/23 | 23PN-136A00068 | 28 | EMM1.0 | M1UK | 100 | 100 | 100 | 100 | . | 100 | . | . | 100 | . | . | . | . | . |
| 496 | 05/23 | 23PN-136A00069 | 28 | EMM1.0 | M1global | 96.76 | 100 | 100 | 100 | . | 100 | . | . | 100 | . | . | . | . | . |
| 497 | 05/23 | 23PN-136A00070 | 28 | EMM1.0 | M1global | 93.2 | 100 | 100 | 100 | . | 100 | . | . | 100 | . | . | . | . | . |
| 498 | 05/23 | 23PN-136A00071 | 28 | EMM1.0 | M1global | 93.2 | 100 | 100 | 100 | . | 100 | . | . | 100 | . | . | . | . | . |
| 499 | 05/23 | 23PN-136A00072 | 82 | EMM92.0 | N/A | . | 91.88 | 100 | . | 100 | 100 | . | . | 100 | . | . | . | . | 100 |
| 500 | 05/23 | 23PN-136A00077 | 28 | EMM1.0 | M1UK | 100 | 100 | 100 | 100 | . | 100 | . | . | 100 | . | . | . | . | . |
| 501 | 05/23 | 23PN-137A00001 | 36 | EMM12.8 | N/A | . | 100 | 100 | . | 100 | 100 | 100 | 100 | . | . | . | . | . | 100 |
| 502 | 05/23 | 23PN-137A00003 | 28 | EMM1.0 | M1global | 93.2 | 100 | 100 | 100 | . | 100 | . | . | 100 | . | . | . | . | . |
| 503 | 05/23 | 23PN-137A00004 | 150 | EMM75.0 | N/A | . | 99.57 | 100 | . | . | 100 | . | . | . | . | 100 | 100 | . | . |
| 504 | 05/23 | 23PN-137A00005 | 433 | EMM49.0 | N/A | . | . | 100 | 100 | . | 100 | 100 | 100 | . | . | . | . | . | . |
| 505 | 05/23 | 23PN-137A00007 | 36 | EMM12.0 | N/A | . | 100 | 100 | . | 100 | 100 | 100 | 100 | . | . | . | . | . | 100 |
| 506 | 05/23 | 23PN-137A00008 | 579 | EMM41.11 | N/A | . | 100 | 100 | . | 100 | 100 | . | . | . | . | 100 | 97.9 | . | 100 |
| 507 | 05/23 | 23PN-137A00012 | 172 | EMM59.0 | N/A | . | 100 | 100 | . | 100 | 100 | . | . | 100 | 100 | . | . | . | 100 |
| 508 | 05/23 | 23PN-137A00016 | 120 | EMM74.0 | N/A | . | 100 | 100 | 100 | 100 | 100 | 98.73 | . | . | . | . | . | . | 100 |
| 509 | 05/23 | 23PN-137A00017 | 172 | EMM59.0 | N/A | . | 100 | 100 | . | 100 | 100 | . | . | 100 | 100 | . | . | . | 100 |
| 510 | 05/23 | 23PN-137A00019 | 55 | EMM2.0 | N/A | . | . | 100 | . | 100 | 100 | . | . | . | 100 | . | . | . | 100 |
| 511 | 05/23 | 23PN-143A00014 | 28 | EMM1.0 | M1global | 93.2 | 100 | 100 | 100 | . | 100 | . | . | 100 | . | . | . | . | . |
| 512 | 05/23 | 23PN-143A00016 | 877 | EMM82.0 | N/A | . | 100 | 100 | . | . | 100 | 100 | 100 | . | . | . | . | . | . |
| 513 | 05/23 | 23PN-143A00017 | 36 | EMM12.8 | N/A | . | 100 | 100 | . | 100 | 100 | 100 | 100 | . | . | . | . | . | 100 |
| 514 | 05/23 | 23PN-143A00018 | 172 | EMM59.0 | N/A | . | 100 | 100 | . | 100 | 100 | . | . | 100 | 100 | . | . | . | 100 |
| 515 | 05/23 | 23PN-143A00019 | 55 | EMM2.0 | N/A | . | . | 100 | . | 100 | 100 | . | . | . | 100 | . | . | . | 100 |
| 516 | 05/23 | 23PN-143A00020 | 36 | EMM12.8 | N/A | . | 100 | 100 | . | 100 | 100 | 100 | 100 | . | . | . | . | . | 100 |
| 517 | 05/23 | 23PN-143A00021 | 579 | EMM41.11~ | N/A | . | 100 | 100 | . | 100 | 100 | . | . | . | . | 100 | 100 | . | 100 |
| 518 | 05/23 | 23PN-143A00023 | 36 | EMM12.8 | N/A | . | 100 | 100 | . | 100 | 100 | 100 | 100 | . | . | . | . | . | 100 |
| 519 | 05/23 | 23PN-143A00024 | 28 | EMM1.0 | M1global | . | 91.88 | 100 | 100 | . | 100 | . | . | 100 | . | . | . | . | . |
| 520 | 05/23 | 23PN-143A00025 | 28 | EMM1.0 | M1UK | . | 85.75 | 100 | 100 | 100 | 100 | . | . | 100 | . | . | . | . | 100 |
| 521 | 05/23 | 23PN-143A00026 | 36 | EMM12.7 | N/A | . | . | 100 | . | 100 | 100 | 100 | 100 | . | . | . | . | . | 100 |
| 522 | 05/23 | 23PN-143A00027 | 28 | EMM1.0 | M1global | . | 100 | 100 | 100 | . | 100 | . | . | 100 | . | . | . | . | . |
| 523 | 05/23 | 23PN-143A00028 | 28 | EMM1.3 | M1UK | 100 | 100 | 100 | 100 | . | 100 | . | . | 100 | . | . | . | . | . |
| 524 | 05/23 | 23PN-143A00029 | 82 | EMM92.0 | N/A | . | 100 | 100 | . | 100 | 100 | . | . | 100 | . | . | . | . | . |
| 525 | 05/23 | 23PN-143A00031 | 28 | EMM1.0 | M1global | 93.2 | 100 | 100 | 100 | . | 100 | . | . | 100 | . | . | . | . | . |
| 526 | 05/23 | 23PN-143A00032 | 36 | EMM12.8 | N/A | . | 92.88 | 100 | . | 100 | 100 | 100 | 100 | . | . | . | . | . | 100 |
| 527 | 05/23 | 23PN-144A00020 | 1176 | EMM28.5 | N/A | . | 94.02 | 100 | . | . | . | . | . | . | . | . | . | . | . |
| 528 | 05/23 | 23PN-150A00004 | 334 | EMM82.0 | N/A | . | 92.17 | 100 | . | . | 100 | 99.58 | 100 | . | . | . | . | . | . |
| 529 | 05/23 | 23PN-150A00006 | 120 | EMM74.0 | N/A | . | 100 | 100 | 100 | 100 | 100 | 98.73 | . | . | . | . | . | . | 100 |
| 530 | 05/23 | 23PN-150A00009 | 28 | EMM1.0 | M1UK | . | 100 | 100 | 100 | 100 | 100 | . | . | 100 | . | . | . | . | 100 |
| 531 | 05/23 | 23PN-150A00011 | 36 | EMM82.0 | N/A | . | 100 | 100 | . | 100 | 100 | 100 | 100 | . | . | 100 | 100 | . | 100 |
| 532 | 05/23 | 23PN-150A00014 | 347 | EMM53.0 | N/A | . | 100 | 100 | . | . | 100 | . | . | . | . | . | . | . | . |
| 533 | 05/23 | 23PN-150A00016 | 28 | EMM1.0 | M1UK | 100 | 100 | 100 | 100 | 100 | 100 | . | . | 100 | . | . | . | . | 100 |
| 534 | 05/23 | 23PN-150A00018 | 579 | EMM41.11 | N/A | . | 100 | 100 | . | 100 | 100 | . | . | . | . | 100 | 100 | . | 100 |
| 535 | 05/23 | 23PN-150A00021 | 82 | EMM92.0 | N/A | . | 100 | 100 | . | 100 | 100 | . | . | 100 | . | . | . | . | 100 |
| 536 | 05/23 | 23PN-150A00022 | 28 | EMM1.0 | M1UK | . | 100 | 100 | 100 | 100 | 100 | . | . | 100 | . | . | . | . | 100 |
| 537 | 05/23 | 23PN-150A00023 | 347 | EMM53.0 | N/A | . | 100 | 100 | . | . | 100 | . | . | . | . | . | . | . | . |
| 538 | 05/23 | 23PN-164A00008 | 28 | EMM1.25 | M1global | 100 | 100 | 100 | 100 | . | 100 | . | . | 100 | . | . | . | . | . |
| 539 | 05/23 | 23PN-157A00001 | 334 | EMM82.0~ | N/A | . | 100 | 100 | . | . | 100 | 100 | 100 | . | . | . | . | . | . |
| 540 | 05/23 | 23PN-157A00002 | 120 | EMM74.0~ | N/A | . | 100 | 100 | 100 | 100 | 100 | 98.73 | . | . | . | . | . | . | 100 |
| 541 | 05/23 | 23PN-157A00003 | 334 | EMM82.0 | N/A | . | 88.18 | 100 | . | . | 100 | 100 | 100 | . | . | . | . | . | . |
| 542 | 05/23 | 23PN-157A00004 | 36 | EMM12.8 | N/A | . | 100 | 100 | . | 100 | 100 | 100 | 100 | . | . | . | . | . | 100 |
| 543 | 05/23 | 23PN-157A00005 | 1176 | EMM28.5 | N/A | . | 100 | 100 | . | . | . | . | . | . | . | . | . | . | . |
| 544 | 05/23 | 23PN-157A00006 | 909 | EMM81.0 | N/A | . | . | 100 | . | . | 100 | 100 | . | . | . | . | . | . | . |
| 545 | 05/23 | 23PN-157A00007 | 28 | EMM1.0 | M1UK | 100 | 87.18 | 100 | . | 100 | 100 | . | . | 100 | . | . | . | . | 100 |
| 546 | 05/23 | 23PN-157A00011 | 242 | EMM12.37 | N/A | . | 100 | 100 | . | 100 | 100 | 100 | 100 | . | . | . | . | . | 100 |
| 547 | 05/23 | 23PN-157A00012 | 36 | EMM12.0 | N/A | . | 100 | 100 | . | 100 | 100 | 100 | 100 | . | . | . | . | 100 | 100 |
| 548 | 05/23 | 23PN-157A00013 | 1176 | EMM28.5 | N/A | . | 100 | 100 | . | . | . | . | . | . | . | . | . | . | . |
| 549 | 05/23 | 23PN-157A00014 | 36 | EMM12.8 | N/A | . | 100 | 100 | . | 100 | 100 | 100 | 100 | . | . | . | . | . | 100 |
